# Metabolomic insights into associations between adiposity markers and liver cancer risk: Results from a prospective cohort study and Mendelian randomization analysis

**DOI:** 10.1371/journal.pmed.1004910

**Published:** 2026-02-02

**Authors:** Zhuo-Ying Li, Hong-Lan Li, Jing Wang, Qiu-Ming Shen, Yi-Xin Zou, Dan-Ni Yang, Yu-Ting Tan, Yong-Bing Xiang

**Affiliations:** 1 State Key Laboratory of System Medicine for Cancer, Shanghai Cancer Institute, Renji Hospital, Shanghai Jiao Tong University School of Medicine, Shanghai, China; 2 Department of Epidemiology, Shanghai Cancer Institute, Shanghai, China; 3 School of Public Health, Fudan University, Shanghai, China; 4 School of Public Health, Shanghai Jiao Tong University School of Medicine, Shanghai, China; National Cancer Institute, UNITED STATES OF AMERICA

## Abstract

**Background:**

The association between adiposity and increased liver cancer risk is well-recognized, yet underlying metabolic mechanisms require elucidation. This study aimed to identify metabolic mediators linking adiposity markers to liver cancer and assess their potential causality using two-sample Mendelian randomization (MR) analysis.

**Methods and findings:**

We conducted a 1:1 matched nested case-control study within a population-based and prospective cohort study—the Shanghai Men’s Health Study (SMHS). The SMHS was initiated in 2002–2006, including 61,469 Chinese men aged 40–74 years, and has been followed up for over 20 years. Targeted metabolomic profiling was performed on baseline plasma samples. Associations between seven anthropometric measurements (body mass index [BMI], waist circumference, waist-to-hip ratio, waist-to-height ratio, a body shape index, hip circumference, and adult weight gain), 186 circulating metabolites, and liver cancer risk were assessed. Linear and conditional logistic regression model adjusted for multiple confounders (including smoking, alcohol drinking, physical activity, chronic hepatitis and cirrhosis, diabetes, etc.) were used. Pathway analysis and network analysis were conducted to explore the biological functions of these metabolites. Parallel mediation analysis was employed to quantify the mediating effects through metabolites. Subsequently, MR analysis was performed to investigate potential causal relationships. This study incorporated 322 incident liver cancer cases and 322 cancer-free controls. Participants diagnosed with liver cancer had higher proportions of seropositive hepatitis B surface antigen (63.7%) compared to their matched controls (6.2%). We identified 27 intermediate metabolites associated with both adiposity markers and liver cancer risk, which formed an interconnected functional network. Pyroglutamic acid demonstrated the most robust consistency, being significantly associated with seven anthropometric measurements (β per doubling with BMI = 0.17; 95% confidence interval [CI]: [0.09, 0.24]) and liver cancer (odds ratio per doubling = 1.56; 95% CI: [1.13, 2.15]). Pathway analysis highlighted significant alterations in energy, lipid, and amino acid metabolism. Specifically, Phenylalanine, tyrosine, and tryptophan biosynthesis showed the highest impact, suggesting a key role for aromatic amino acid metabolism. Parallel mediation analysis demonstrated significant indirect effects via intermediate metabolites for six of the seven anthropometric measurements, with the proportion mediated by the identified metabolite clusters reaching 0.16 (95% CI: [0.05, 0.29]) for BMI. MR analysis provided evidence supporting potential causality for 23 of 108 initially observed associations. The strongest association was observed between WC and oxoglutaric acid (β_IVW_ per standard deviation = 0.31; 95% CI: [0.17, 0.43]). Notably, while the observational analysis suggested a broad metabolic mediation of the adiposity marker-liver cancer association, the MR findings pinpointed a more specific and limited set of causal metabolic mediators. The main limitation of this study was the population mismatch between the observational (Chinese men) and the MR (European ancestry) analyses, which may limit the generalizability of the findings to other populations.

**Conclusions:**

Integrating prospective observational and genetic evidence, we identified specific metabolic mediators linking adiposity to liver cancer, particularly involving amino acid, lipid and energy metabolism. These findings enhanced molecular understanding of adiposity-driven hepatocarcinogenesis and provided potential metabolic targets for future primary prevention strategies.

## Author summary

**Why was this study done?:**

- While it is well-known that adiposity increases liver cancer risk, the specific biological mechanisms connecting them are not fully understood.
- This study was conducted to identify the key circulating metabolites that intermediate the association between adiposity markers and liver cancer and to assess whether these links are causal.

**What did the researchers do and find?:**

- We comprehensively analyzed metabolomics data from a nested case-control study of 322 liver cancer cases and 322 matched controls within the prospective Shanghai Men’s Health Study.
- We identified 27 specific metabolites, primarily involved in energy, lipid, and amino acid metabolism, that were associated with both various adiposity markers and liver cancer risk.
- To investigate causality, we then applied a Mendelian randomization (MR) approach, which provided genetic evidence supporting a potential causal role of 21 associations between adiposity markers and metabolites, characterized primarily by elevated levels of adiposity markers and specific amino acids and organic acids (e.g., creatine, tyrosine, and 2-hydroxybutyric acid).

**What do these findings mean?:**

- By integrating observational data with genetic evidence, our findings offer new molecular insights into how adiposity may drive the development of liver cancer, highlighting specific dysregulated metabolic pathways.
- These identified metabolites could represent potential new biomarkers for early risk detection or targets for future prevention strategies aimed at breaking the link between adiposity and liver cancer.
- While our findings from a specific cohort of Chinese men require validation in other populations, they significantly advance the understanding of liver carcinogenesis and suggest new avenues for public health interventions.

## Abstract 3

Zhuo-Ying Li and colleagues identify metabolic mediators linking adiposity markers to liver cancer in a nested case–control study, then assess their potential causality using a two-sample Mendelian randomization approach.

## Introduction

Primary liver cancer is the sixth most commonly diagnosed cancer and the third leading cause of cancer-related death worldwide, with approximately 865,269 new cases and 757,948 deaths reported in 2022 [1]. Chronic infections with hepatitis B virus (HBV) and hepatitis C virus infections are major risk factors for liver cancer [2]. With the widespread use of HBV vaccination and direct-acting antiviral therapies for hepatitis C virus, the proportion of liver cancer cases attributable to viral factors is expected to gradually decrease, while the proportion attributable to metabolic factors, such as excess body fatness, type 2 diabetes mellitus (T2DM), and metabolic dysfunction-associated steatotic liver disease, is expected to increase [3,4].

It is well established that excess body fatness, typically measured by anthropometric measurements such as body mass index (BMI) and waist circumference (WC), is associated with an increased risk of liver cancer [4,5]. Several studies, including one by our team, have shown that weight gain during adulthood is also associated with an increased risk of liver cancer [6,7]. Mendelian randomization (MR) studies also supported the causal relationship between BMI and liver cancer risk [8,9]. Despite extensive evidence supporting the association between adiposity markers and liver cancer risk, the understanding of the biological mechanisms linking them remains limited [4,10]. Several hypotheses have emerged, including alterations in insulin signaling, dysregulation of adipocytokines, chronic low-grade inflammation, and sex hormone metabolism [5,10–12]. The alterations in gut microbiome and gut hormones may also play important roles [10,12].

As the end products of biochemical reactions in organisms, metabolites provide a comprehensive reflection of gene expression, protein function, and environmental interactions. The application of high-throughput metabolomics technology has facilitated the identification of numerous circulating metabolites associated with excess body fatness and liver cancer, respectively. For example, Cirulli and colleagues identified and validated 49 metabolites, predominantly lipids and amino acids, that were significantly associated with BMI using serum samples collected at three time points from over 1,200 European individuals [13]. An untargeted metabolomic analysis of 1,534 postmenopausal women identified 260 metabolites significantly associated with BMI and 218 with WC, including amino acids, acyl-carnitines, glycerophospholipids, sphingolipids, nucleotides, and bile acids [14]. With regard to liver cancer, existing prospective studies have shown that amino acids, fatty acids, bile acids, and organic acids constitute the major classes of metabolites associated with the risk of the disease [15,16].

In accordance with the “meet-in-the-middle” framework proposed by Vineis and colleagues, identifying intermediate biomarkers that link environmental exposures to health outcomes can illuminate the biological pathways through which exposures contribute to disease [17,18]. We propose that specific metabolites associated with both adiposity markers (exposure) and liver cancer (outcome) might serve as key mediators. Investigation of these metabolites and their related biochemical pathways may provide valuable insights into the underlying biological mechanisms between adiposity and increased liver cancer risk [19]. On this basis, MR analysis can be further conducted to assess the potential causal relationships between adiposity markers, metabolites, and liver cancer, as it addresses some important limitations of observational epidemiological studies, such as confounding and reverse causation [20]. Such a triangulation approach, integrating evidence from different epidemiological approaches with different major sources of potential bias, may strengthen the causal inference between adiposity, metabolites and liver cancer [21].

Using the data from a large prospective cohort study—the Shanghai Men’s Health study (SMHS), we have previously confirmed the association between several anthropometric measurement and liver cancer risk [22]. This finding is further supported by even stronger and more consistent associations observed in the parallel Shanghai Women’s Health Study, drawn from the same source population [7]. In this study, we utilized the metabolomic profiling data from a case-control study nested within the SMHS, to identify potential intermediate metabolic biomarkers linking adiposity markers to liver cancer risk via a “meet-in-the-middle” strategy. To address the issues of confounding and reverse causation, the associations observed in the nested case-control study were further validated using two-sample MR approach.

## Methods

This study was conducted in accordance with both the Declarations of Helsinki and Istanbul. Approval was granted by the Ethics Committee of the Renji Hospital Ethics Committee of Shanghai Jiao Tong University School of Medicine (KY2021-029). All analyses were conducted with R version 4.4.1 (R Core Team, Vienna, Austria). A 2-sided *P* value of less than 0.05 was considered statistically significant unless noted otherwise. This study was reported in accordance with the Strengthening the Reporting of Observational Studies in Epidemiology (STROBE) and the STROBE-MR Statement [23,24] (Tables A and B in S1 Checklist).

### Methods of the nested case-control study

#### Study design.

**Fig 1:**
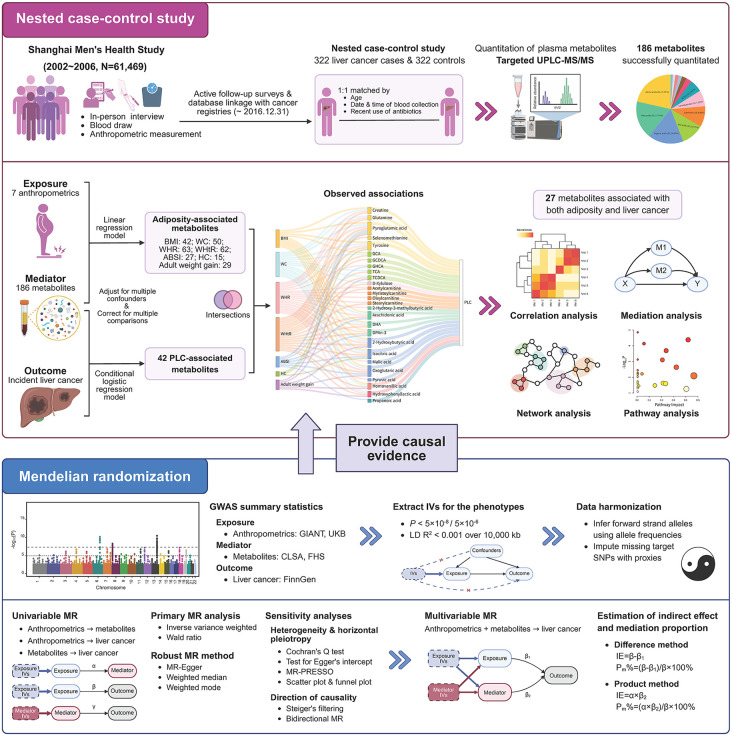
Study design and workflow. This figure outlines the overall study design. Using metabolomic profiling data from a nested case-control study within the SMHS, a “meet-in-the-middle” strategy was employed to identify potential intermediate metabolites linking seven anthropometric measurements to liver cancer risk. Then a two-sample MR approach was used to provide causal evidence for observed associations. The figure was created in https://BioRender.com. BMI, body mass index; WC, waist circumference; WHR, waist-to-hip ratio; WHtR, waist-to-height ratio; ABSI, a body shape index; HC, hip circumference; PLC, primary liver cancer; MR, Mendelian randomization; GIANT, Genetic Investigation of ANthropometric Traits; UKB, UK Biobank; CLSA, Canadian Longitudinal Study on Aging; FHS, Framingham Heart Study; LD, linkage disequilibrium; SNP, single-nucleotide polymorphism; IV, instrument variable; IE, indirect effect.

The present nested case-control study was conducted based on the SMHS, the rationale for which has been published elsewhere [25]. Written informed consent was obtained from each individual participant included in the cohort. Briefly, the SMHS is a population-based and prospective cohort initiated in 2002–2006, including 61,469 Chinese men aged 40–74 years. Demographic characteristics, medical history, dietary habits, physical activity participation, and other lifestyle factors of the study participants were asked at baseline interview. Anthropometric measurements and blood samples were also collected following standardized methods and protocols. The present nested case-control study included 322 incident liver cancer cases and their 1:1 matched controls [15]. The matching factors included age at blood collection (±2 years), date of blood collection (±30 days), time of blood collection (morning/afternoon), and antibiotics use during the preceding week (yes/no). The study design and workflow are outlined in Fig 1 and detailed in S1 Text.

#### Anthropometric measurements.

Anthropometrics including height, weight, WC, and hip circumference (HC) of the cohort members were recorded according to a standardized protocol at the end of the baseline interview [22,25]. Additionally, weight at 20 years old were obtained by a self-administrated questionnaire. To better characterize different types of fat accumulation, seven exposure variables were used to indicate overall obesity, abdominal obesity, gluteofemoral obesity, and weight gain through adulthood: BMI, WC, waist-to-hip ratio (WHR), waist-to-height ratio (WHtR), a body shape index (ABSI), HC, and adult weight gain. The definition of the exposure variables is shown in Table A in S1 Appendix.

#### Quantitation of plasma metabolites.

The quantitation of plasma metabolites for 322 liver cancer cases and 322 controls was performed using an ultraperformance liquid chromatography coupled to tandem mass spectrometry (UPLC–MS/MS) system (ACQUITY UPLC-Xevo TQ-S, Waters Corp., Milford, MA, USA). After standard quality control procedures, the absolute concentrations (μmol/L) of 186 known metabolites were quantified. Log_2_-transformation was applied to address the skewed distribution of metabolite concentrations. Details of the experimental procedures can be found in previous publications [15,26] and the S1 Text.

#### Statistical analyses.

Baseline characteristics of the study participants were presented as frequency with proportion for categorical variables, as mean with standard deviation (SD) for continuous variables with approximately normal distribution, and as median with 25th and 75th percentiles (*Q*1 and *Q*3) for skewed variables. The distribution of anthropometric measurements was described using min, max, mean, SD, median, 25th, and 75th percentiles.

To identify metabolites associated with both anthropometric measurements and liver cancer risk, we utilized a two-step strategy. First, we used linear regression models to examine the associations between anthropometric measurements (explanatory variables) and plasma metabolites (dependent variables), adjusting for age, education level, personal income, dietary habits, cigarette smoking, alcohol drinking, and physical activity. Second, we fitted conditional logistic regression models to examine the association between plasma metabolites and liver cancer risk, adjusting for age, dietary habits, cigarette smoking, alcohol drinking, BMI, physical activity, chronic hepatitis and cirrhosis, medical history of cholelithiasis, and medical history of T2DM. Next, we intersected the results from steps 1 and 2 to identify metabolites associated with both anthropometric measurements and liver cancer risk. Several sensitivity analyses were conducted to examine the robustness of the main analysis and the details can be found in S1 Text. Potential confounders were selected a priori based on their known associations with anthropometrics and liver cancer and their potential to affect plasma metabolites. We created directed acyclic graphs to determine the minimal sufficient adjustment sets for the associations [27] (Figs A and B in S2 Appendix). The Benjamini-Hochberg false discovery rate (FDR) was applied to account for multiple comparisons across 186 metabolites [28]. Associations with an FDR less than 0.05 were retained. Restricted cubic spline (RCS) functions were used to capture potential non-linear relationships [29,30]. If the nonlinearity test for the RCS terms of an exposure variable was statistically significant (*P*_non-linear_ < 0.05), the association was retained for subsequent analyses. We estimated the pairwise correlations of metabolites associated with both anthropometric measurements and liver cancer risk by calculating the partial Spearman rank correlation coefficients (Spearman’s *ρ*), adjusted for age and fasting time. To avoid potential collider bias, the correlation analyses were performed on the controls only.

To explore the associations between potential intermediate metabolites, we generated a data-driven network. The skeleton of the network was constructed using the conditional independence-based PC algorithm [31,32]. The walktrap algorithm was then utilized to identify densely connected metabolite modules within the network [33,34].

To identify the most relevant biochemical pathways linking adiposity markers to liver cancer, we conducted pathway enrichment analysis and pathway topology analysis using the MetaboAnalyst version 6.0 (https://www.metaboanalyst.ca/) [35]. The Kyoto Encyclopedia of Genes and Genomes (KEGG) Homo sapiens database and the Small Molecule Pathway DataBase (SMPDB) 2.0 were selected as the pathway libraries [36,37]. We selected the global test as the quantitative enrichment method and the relative betweenness centrality as the node importance measure for topology analysis. The Human Metabolome Database IDs were used as standard metabolite names [38]. The iPATH website tool was utilized to visualize the identified KEGG pathways and relevant metabolites [39].

A parallel multiple mediation analysis was conducted based on metabolites associated with both anthropometric measurements and liver cancer risk. The PROCESS R macro (version 4.3) developed by Hayes and colleagues was utilized to estimate the total effect and direct effect of anthropometric measurements on liver cancer, as well as the indirect effect (IE) of anthropometric measurements on liver cancer through intermediate metabolites [40]. Age, education level, personal income, dietary habits, cigarette smoking, alcohol drinking, physical activity, chronic hepatitis and cirrhosis, medical history of cholelithiasis, and medical history of T2DM were included in the model as covariates. Considering that the PROCESS macro support only 10 mediators, hierarchical clustering was performed on the mediators prior to the mediation analysis [41]. The first principal component derived from each cluster was subsequently included in the models as a mediator.

### Methods of the two‑sample Mendelian randomization

We conducted two-sample MR analysis to provide extra causal evidence for significant associations observed in the nested case-control study. Genome-wide association study (GWAS) summary statistics from non-overlapping samples for anthropometric measurements, metabolites, and liver cancer were obtained, as detailed in Table A in S3 Appendix and S1 Text. Briefly, the summary statistics were obtained from the Genetic Investigation of ANthropometric Traits (GIANT) consortium, the UK Biobank (UKB), the Canadian Longitudinal Study on Aging (CLSA) cohort, the Framingham Heart Study (FHS), and the FinnGen study [42–50]. All included GWAS were conducted on individuals of European descent. We did not find available GWAS summary data for WHtR, adult weight gain, selenomethionine, and D-xylulose. Therefore, these traits were not included in the MR analysis.

The selection of instrument variable (IV) was guided by three core assumptions of MR: relevance, independence, and exclusion restriction. For anthropometric measurements and hepatocellular carcinoma (HCC) (in bidirectional MR), all single-nucleotide polymorphisms (SNPs) associated with the exposure at a genome-wide significance threshold (*P* < 5 × 10^−8^) were selected as IVs. For metabolites, a less stringent threshold (*P* < 5 × 10^−6^) was used due to relatively small sample sizes of the CLSA (~8,000) and FHS (2,076) GWAS datasets. The resulting SNPs were pruned with a linkage disequilibrium (LD) threshold of r^2^ < 0.001 within a 10,000-kilobase window. The exposure and outcome datasets were then harmonized to ensure that the effect of each SNP corresponded to the same allele.

The primary univariable MR analysis was conducted using the multiplicative random-effects inverse-variance weighted (IVW) method [51]. To assess the robustness of the main findings, the MR-Egger [52], weighted median [53], and weighted mode [54] methods were additionally applied. When only one SNP remained as a valid IV, the Wald ratio method was used to calculate the causal estimates [55]. In addition to employing robust MR methods, various sensitivity analyses were performed to assess potential violations of MR assumptions and to validate the causal associations identified by the IVW method. Further details regarding the sensitivity analyses are provided in the S1 Text.

To further investigate the independent causal effects of adiposity markers and metabolites on the risk of liver cancer, and to investigate the potential mediating role of metabolites in the association between adiposity markers and liver cancer risk, we performed multivariable MR analysis [56]. The indirect (mediated) effect through metabolites was then examined using two approaches: the Difference method and the Product method (Fig 1 and S1 Text) [57]. The mediation proportion was calculated as the ratio of the indirect effect to the total effect.

## Results

### Results of nested case-control study

Baseline characteristics of the study participants are presented in Table 1. Participant diagnosed with liver cancer had higher proportions of seropositive hepatitis B surface antigen (HBsAg) (63.66%), a medical history of chronic hepatitis (26.40%), cirrhosis (12.73%), cholelithiasis (14.29%), and a family history of liver cancer (9.32%) compared to their matched controls (6.21%, 3.42%, 0.31%, 6.52%, and 3.11%, respectively). The median age at diagnosis for liver cancer cases was 65.03 years (*Q*1 = 55.85, *Q*3 = 73.80), and the median interval between sample collection and diagnosis was 5.89 years (*Q*1 = 3.02, *Q*3 = 8.90).

**Table 1:** Baseline characteristics of the study participants^a^.

	Liver cancer cases (*N* = 322)	Matched controls (*N* = 322)
Age at blood collection (years old)	57.57 [50.66; 68.31]	57.01 [50.66; 68.59]
Fasting time (hours)		
<3	79 (24.53%)	76 (23.60%)
3–6	165 (51.24%)	166 (51.55%)
≥6	78 (24.22%)	80 (24.84%)
Education		
Elementary school or less	34 (10.56%)	35 (10.87%)
Middle school	243 (75.47%)	219 (68.01%)
College or above	45 (13.98%)	68 (21.12%)
Income (Yuan/month)		
<1,000	213 (66.15%)	175 (54.35%)
1,000–2,999	84 (26.09%)	115 (35.71%)
≥3,000	25 (7.76%)	32 (9.94%)
Ever smoker	232 (72.05%)	203 (63.04%)
Ever drinker	109 (33.85%)	105 (32.61%)
Hepatitis B virus infection^b^	205 (63.66%)	20 (6.21%)
History of chronic hepatitis	85 (26.40%)	11 (3.42%)
History of cirrhosis	41 (12.73%)	1 (0.31%)
History of cholelithiasis	46 (14.29%)	21 (6.52%)
History of type 2 diabetes mellitus	33 (10.25%)	27 (8.39%)
Family history of liver cancer	30 (9.32%)	10 (3.11%)
Total physical activity (MET-hour/week)	58.13 [36.70; 83.41]	58.07 [35.43; 85.43]
Chinese Food Pagoda score	30.36 [26.91; 34.27]	30.72 [27.33; 33.78]
BMI (kg/m^2^)	23.79 ± 3.44	23.53 ± 3.02
WC (cm)	85.57 ± 9.45	84.83 ± 8.58
WHR	0.90 ± 0.06	0.90 ± 0.06
WHtR	0.51 ± 0.06	0.50 ± 0.05
ABSI (kg^-2/3^m^11/6^)	0.08 [0.08; 0.08]	0.08 [0.08; 0.08]
HC (cm)	94.50 [90.00; 99.00]	94.00 [90.00; 98.00]
Adult weight gain (kg)^c^	11.44 ± 9.70	10.91 ± 9.43

^a^Continuous variables were presented as mean ± standard deviation (SD) for variables with approximate normal distribution, and as median [*Q*1; *Q*3] for skewed variables; categorical variables were presented as frequency (percentages).

^b^Four cases and two controls lacked this information. Their self-reported history of chronic hepatitis was used as a proxy.

^c^*N* = 559 because self-reported data on weight at age 20 were unavailable for 46 cases and 39 controls.

Abbreviations: MET, metabolic equivalent of task; BMI, body mass index; WC, waist circumference; WHR, waist-to-hip ratio; WHtR, waist-to-height ratio; ABSI, a body shape index; HC, hip circumference.

**Fig 2:**
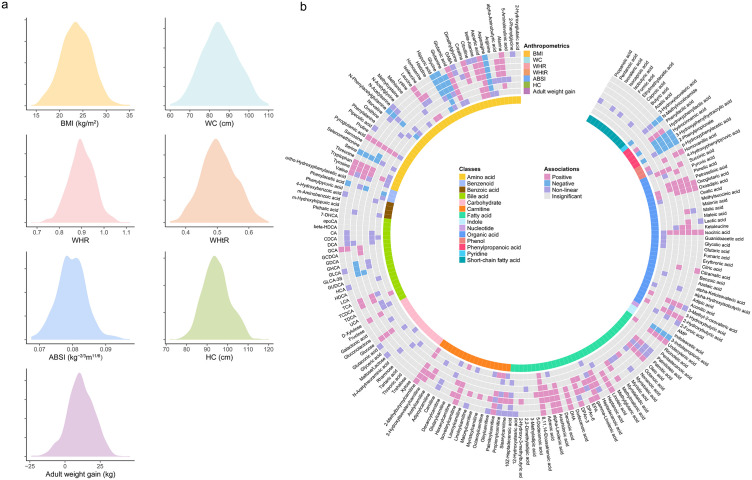
Associations between seven anthropometric measurements and plasma metabolite concentrations. **a** Density plots illustrating the distribution of the seven anthropometric measurements. **b** Circular visualization summarizing the results of linear regression models examining the associations between the seven anthropometric measurements and 186 plasma metabolites. The color of each tile indicates the category of association, determined by the regression coefficient (*β*), FDR, and *P*_non-linear_: positive (pink; *β* > 0 and FDR < 0.05), negative (blue; *β* < 0 and FDR < 0.05), non-linear (purple; *P*_non-linear_<0.05), and insignificant (gray; FDR ≥ 0.05 and *P*_non-linear_≥0.05). The innermost colored ring segments indicate the chemical class of each metabolite. All linear regression models were adjusted for age, education level, personal income, dietary habits, cigarette smoking, alcohol drinking, and total physical activity. BMI, body mass index; WC, waist circumference; WHR, waist-to-hip ratio; WHtR, waist-to-height ratio; ABSI, a body shape index; HC, hip circumference.

The distribution of seven anthropometric measurements is presented in Fig 2a and Table B in S1 Appendix. The Spearman rank correlation coefficients between the seven anthropometric measurements are shown in Table C in S1 Appendix. The correlations between ABSI and BMI (*ρ* = −0.03) and ABSI and adult weight gain (*ρ* = 0.07) were weak and statistically insignificant. All other anthropometric measurements were significantly correlated with each other, with WC and WHtR showing the highest correlation (*ρ* = 0.94).

The associations between anthropometric measurements and plasma metabolites are presented in Fig 2b and Tables D–J in S1 Appendix. We observed 59, 65, 74, 67, 50, 27, and 52 metabolites associated with BMI, WC, WHR, WHtR, ABSI, HC, and adult weight gain, respectively. Based on the risk estimates (β) and statistical significance (FDR and *P*_non-linear_), we categorized the associations into four groups: positive, negative, non-linear, and insignificant. Linear associations were more common than non-linear ones, with the majority being positive and a few showing negative associations (Fig 2b). Seven metabolites were consistently associated with all anthropometric measurements, including asparagine, aspartic acid, glutamic acid, glycine, pyroglutamic acid, 8,11,14-eicosatrienoic acid, and isocitric acid. Sensitivity analyses substantially supported the main results (Tables D–J in S1 Appendix). In the sensitivity analyses, 42, 50, 63, 62, 27, 15, and 29 associations were replicated for BMI, WC, WHR, WHtR, ABSI, HC, and adult weight gain, respectively. Finally, 92 metabolites that were associated with at least one anthropometric measurement were retained for further analysis.

**Fig 3:**
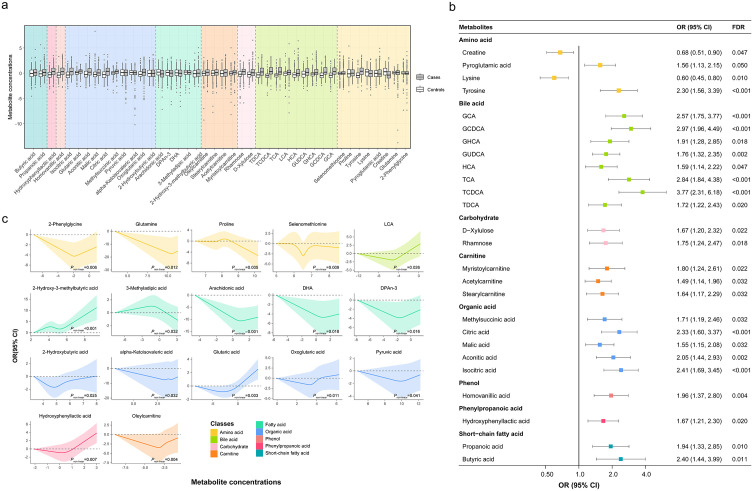
Conditional logistic regression model identified 42 metabolites associated with liver cancer risk. **a** Comparison of Z-score normalized concentrations for the 42 metabolites between liver cancer cases (darker boxes) and matched controls (lighter boxes). Metabolites are grouped and background-colored by biochemical class. Boxes represent the interquartile range, and the central line is the median. **b** ORs per doubling and 95% CIs for 26 metabolites exhibiting a significant linear association (FDR < 0.05). Points are colored by metabolite class. **c** Dose-response curves for 17 metabolites showing a significant non-linear association (*P*_non-linear_ <0.05) with liver cancer. All conditional logistic regression models were adjusted for age, dietary habits, cigarette smoking, alcohol drinking, BMI, physical activity, chronic hepatitis and cirrhosis, medical history of cholelithiasis, and medical history of T2DM. One metabolite (hydroxyphenyllactic acid) meets criteria for both linear (FDR < 0.05) and non-linear (*P*_non-linear_ <0.05) significance and thus appears in both panels (**b**) and **(c)**. OR, odds ratio; CI, confidence interval; FDR, false discovery rate.

Conditional logistic regression models identified 42 metabolites that were significantly associated with liver cancer risk after adjusted for multiple confounders and corrected for multiple comparisons (Fig 3 and Table K in S1 Appendix). The distribution of metabolite concentrations for liver cancer cases and control groups are shown in Fig 3a. Among the 26 metabolites showing a linear association with liver cancer, 24 of them were positively associated with liver cancer, except for creatine (odds ratio [OR] = 0.68, 95% CI: [0.51, 0.90], *P* = 0.006) and lysine (OR = 0.60, 95% CI: [0.45, 0.80], *P* < 0.001) (Fig 3b). Potential nonlinear relationships were observed for 17 metabolites and liver cancer risk, with a *P*_non-linear_ of less than 0.05 (Table K in S1 Appendix). The dose-response curves for these metabolites are presented in Fig 3c. Of note, hydroxyphenyllactic acid met the criteria for both linear and non-linear significance and thus appears in both figures. (Fig 3b and 3c). Sensitivity analyses excluding participants with HBV infection or chronic liver disease yielded results largely consistent with the main analysis (Tables K in S1 Appendix), indicating that the identified metabolic associations were not driven by viral hepatitis status.

**Fig 4:**
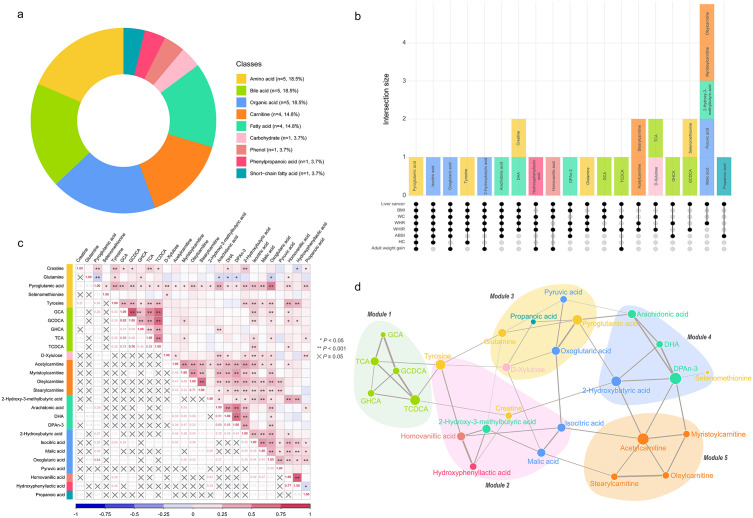
Meet-in-the-middle approach identified 27 intermediate metabolites linking adiposity markers to liver cancer risk. **a** Distribution of the 27 intermediate metabolites by biochemical class. **b** UpSet plot illustrating the intersection of metabolites associated with liver cancer and various combinations of anthropometric measurements. The bottom matrix (connected dots) indicates the specific combination of variables, while the top vertical bars represent the number (intersection size) and names of metabolites shared exclusively by that combination. Bars are colored by metabolite class. **c** Partial correlation matrix for the 27 intermediate metabolites (adjusted for age and fasting time, analysis conducted in controls only). *P* values for partial correlations are presented in Table N in S1 Appendix. **d** Data-driven network of the 27 intermediate metabolites. Modules were determined by the walktrap algorithm. Node size is proportional to degree centrality, and edge thickness represents the strength of partial correlations. Identified modules generally clustered metabolites from the same or metabolically closely related classes. BMI, body mass index; WC, waist circumference; WHR, waist-to-hip ratio; WHtR, waist-to-height ratio; ABSI, a body shape index; HC, hip circumference; PLC, primary liver cancer.

Integrating results from the linear regression and conditional logistic regression models, we derived 27 metabolites that potentially mediate the association between adiposity markers and liver cancer risk. These metabolites represented diverse chemical classes, predominantly amino acids (*n* = 5, 18.5%), bile acids (*n* = 5, 18.5%), organic acids (*n* = 5, 18.5%), carnitines (*n* = 4, 14.8%), and fatty acids (*n* = 4, 14.8%) (Fig 4a). To disentangle the shared and specific metabolic signatures across different adiposity markers, we utilized an UpSet plot (Fig 4b). This plot revealed that most identified metabolites were associated with multiple anthropometric measurements, suggesting a shared metabolic basis for different patterns of fat accumulation. Specifically, pyroglutamic acid demonstrated the most robust consistency, being significantly associated with all seven anthropometric measurements and liver cancer. Furthermore, a cluster of metabolites, including isocitric acid, oxoglutaric acid, and tyrosine, showed broad consistency across most anthropometric measurements (associated with 5 or 6 out of 7 indices). Conversely, other metabolites exhibited distinct patterns linking specific subsets of anthropometric measurements to liver cancer risk. Pairwise correlations among these intermediate metabolites varied substantially (Fig 4c), with the strongest correlation observed between GCA and GCDCA (*ρ*_partial_ = 0.82).

The data-driven network analysis further elucidated the functional organization of these metabolites (Fig 4d). The walktrap algorithm identified five densely connected modules, which generally grouped metabolites from biological related classes. For example, Module 1 comprised bile acids, Module 5 consisted of carnitines, and Module 2 was composed mainly of amino acid derivatives. The predominant metabolites for Modules 1–5 were TCDCA, tyrosine, pyroglutamic acid, DPAn-3, and acetylcarnitine, respectively (Fig 4d).

**Fig 5:**
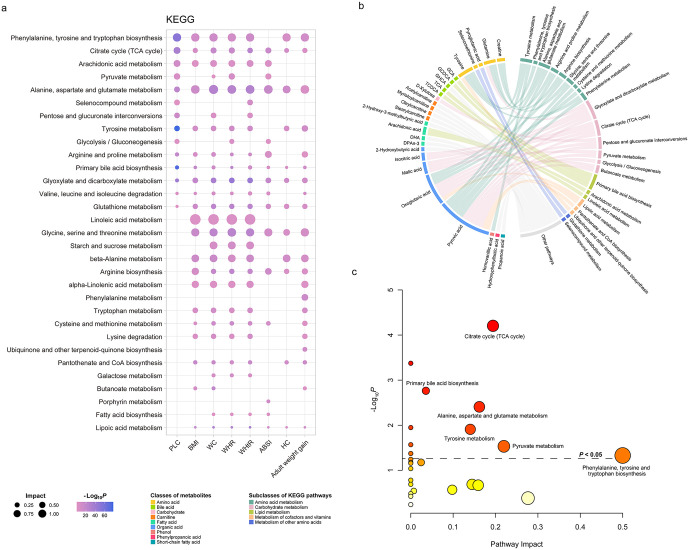
Enriched KEGG pathways associated with adiposity markers and liver cancer risk, and their connection to intermediate metabolites. **a** Quantitative pathway analysis showing pathways associated with liver cancer and seven anthropometric measurements based on the KEGG database. Bubble size indicates the pathway Impact score, and color intensity represents enrichment significance (−Log_10_ *P* value). **b** Chord diagrams illustrating the associations between the 27 identified intermediate metabolites and the significantly enriched KEGG pathways. Colors denote metabolite classes and pathway subclasses. Links connect metabolites to the pathways they are involved in. **c** Qualitative pathway enrichment analysis (hypergeometric test) and topology analysis of the 27 intermediate metabolites based on KEGG database. Results are displayed as bubble plots where the x-axis represents the pathway impact score (calculated based on pathway topology) and the y-axis represents the enrichment significance (−log10 *P* value). Bubble size is proportional to the pathway impact, and color intensity corresponds to the enrichment *P* value. The dashed line indicates the significance threshold (*P* = 0.05). BMI, body mass index; WC, waist circumference; WHR, waist-to-hip ratio; WHtR, waist-to-height ratio; ABSI, a body shape index; HC, hip circumference; PLC, primary liver cancer; KEGG, Kyoto Encyclopedia of Genes and Genomes.

Quantitative pathway analyses revealed multiple important biochemical pathways linking adiposity markers to liver cancer (Fig 5 and Fig A in S4 Appendix and Tables L and M in S1 Appendix). Using the KEGG database as pathway library, Phenylalanine, tyrosine, and tryptophan biosynthesis showed the highest impact value and was enriched across liver cancer and 6 anthropometric measurements (Fig 5a and Table L in S1 Appendix). Other highly enriched pathways included Alanine, aspartate, and glutamate metabolism and Tyrosine metabolism. To visualize the interplay between specific metabolites and these pathways, we constructed a chord diagram (Fig 5b), which highlights how the 27 intermediate metabolites map to key metabolic processes. For instance, organic acids such as pyruvic acid and oxoglutaric acid were central to multiple enriched pathways. Consistent with the quantitative results, the qualitative pathway analysis (integrating enrichment and topology analyses) also identified Phenylalanine, tyrosine, and tryptophan biosynthesis and Citrate cycle (TCA cycle) as the most impactful pathways (Fig 5c). We also performed parallel pathway analyses using the SMPDB database to validate these findings. The results from SMPDB were largely consistent with those from KEGG, identifying biologically relevant pathways such as the Malate-aspartate shuttle. These additional results are presented in Fig A in S4 Appendix and Table M in S1 Appendix.

**Fig 6:**
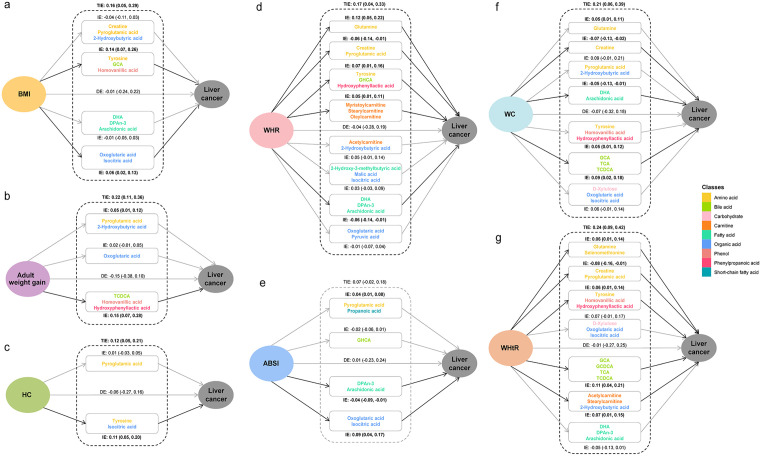
Results of mediation analysis showing metabolite clusters as mediators linking anthropometric measurements to liver cancer risk. Panels illustrate mediation results for each anthropometric measurement as the exposure: **a** BMI. **b** Adult weight gain. **c** HC. **d** WHR. **e** ABSI. **f** WC. **g** WHtR. Due to analytical limitations (PROCESS macro supports ≤10 mediators), potential metabolite mediators were first grouped using hierarchical clustering. The first principal component derived from each cluster was then used as the mediator variable representing that cluster in the models. Metabolites belonging to each cluster are listed within the boxes. Paths represent estimated effects: arrows from anthropometric measurements to liver cancer show the direct effect; arrows involving mediator clusters represent indirect effect. The specific indirect effect mediated through each cluster is shown, along with the total indirect effect across all mediator clusters for a given exposure. Effect estimates with 95% CIs are provided for direct effect, indirect effect, and total indirect effect. Statistical significance is determined by whether the bootstrap CIs excludes the null value. All models were adjusted for age, education level, personal income, dietary habits, cigarette smoking, alcohol drinking, physical activity, chronic hepatitis and cirrhosis, medical history of cholelithiasis, and medical history of T2DM. BMI, body mass index; WC, waist circumference; WHR, waist-to-hip ratio; WHtR, waist-to-height ratio; ABSI, a body shape index; HC, hip circumference; DE, direct effect; IE, indirect effect; TIE, total indirect effect.

Parallel mediation analyses indicated that the direct effect of all seven anthropometric measurements on liver cancer risk were statistically insignificant (Fig 6). Conversely, the total IE mediated through metabolites were significant for six anthropometric measurements, with effect size ranging from 0.12 (95% CI: [0.05, 0.21]) to 0.24 (95% CI: [0.09, 0.42]). Notably, while the total IE of ABSI was insignificant (0.07, 95% CI: [−0.02, 0.19]), two distinct metabolite clusters demonstrated significant IEs in opposite directions: a cluster comprising two fatty acids (DPAn-3 and arachidonic acid) exhibited a negative IE (−0.04, 95% CI: [−0.09, −0.01]), whereas a cluster of two organic acids (oxoglutaric acid and isocitric acid) showed a positive IE (0.09, 95% CI: [0.04, 0.17]) (Fig 6e). Similar bidirectional IEs among different metabolite clusters were observed in the association between other anthropometric measurements and liver cancer, highlighting the complexity of the metabolic alterations in the development of liver cancer. The hierarchical clustering results for intermediate metabolites are presented in Figs A–G in S5 Appendix.

### Results of two‑sample Mendelian randomization

**Fig 7:**
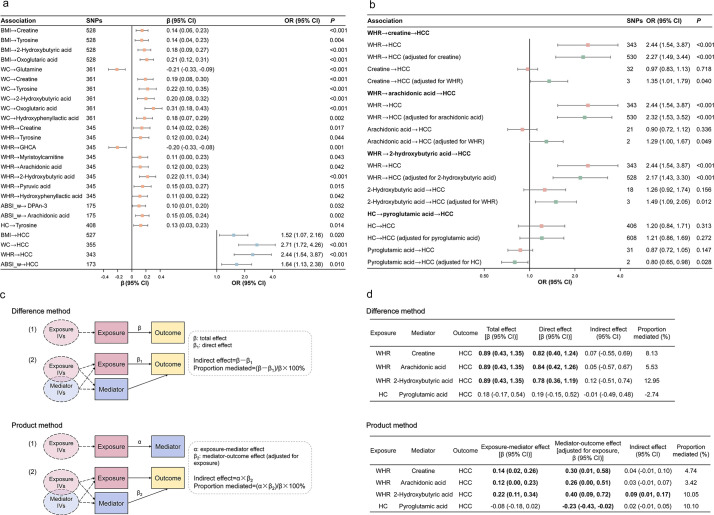
Two-sample MR analysis investigating causal pathways from adiposity markers to liver cancer via intermediate metabolites. **a** Forest plot summarizing statistically significant associations identified through univariable MR. Causal estimates are presented as β or OR per SD increase in exposure with 95% CIs. **b** Forest plot summarizing statistically significant results from multivariable MR. **c** Diagram illustrating the Product method and the Difference method for the estimation of indirect effect and mediation proportion. **d** Mediation MR results showing estimated total, direct, indirect effects, and proportion mediated (%) using the Difference and the Product methods. BMI, body mass index; WC, waist circumference; WHR, waist-to-hip ratio; ABSI, a body shape index; HC, hip circumference; IV, instrument variable; HCC, hepatocellular carcinoma; SNP, single-nucleotide polymorphism; OR, odds ratio; CI, confidence interval.

The results of the univariable MR analysis are summarized in Fig 7a and detailed in Tables B and C in S3 Appendix. The primary IVW method identified 21 potential causal associations between anthropometric measurements and circulating metabolites. The strongest association was observed between WC and oxoglutaric acid (β_IVW_ per SD = 0.31, 95% CI: [0.17, 0.43], *P* < 0.001). Most identified associations were positive, except for WC → glutamine (β_IVW_ per SD = −0.21, 95% CI: [−0.33, −0.09], *P* < 0.001) and WHR → GHCA (β_IVW_ per SD = −0.20, 95% CI: [−0.33, −0.08], *P* = 0.001). The univariable MR also supported the causal associations between BMI, WC, WHR, ABSI (women only), and HCC. For the majority of significant associations identified by IVW, the results from the MR-Egger, weighted median, and weighted mode methods were consistent with the IVW results, demonstrating effect estimates in the same direction (Tables B and C in S3 Appendix). Exceptions included WC → creatine, WHR → pyruvic acid, and WHR → hydroxyphenyllactic acid, where the ME-Egger or weighted mode methods produced non-significant estimates in the opposite direction.

Sensitivity analyses largely corroborated the observed causal associations with some exceptions. Specifically, the Cochran’s Q test and MR-PRESSO global test found evidence of heterogeneity and horizontal pleiotropy in WHR → creatine, and WHR → HCC associations (Tables D and E in S3 Appendix). Nevertheless, these associations remained statistically significant after removing outlier variants identified by MR-PRESSO (WHR → creatine: β_IVW_ per SD = 0.13, 95% CI: [0.02, 0.25], *P* = 0.024; WHR → HCC: OR_IVW_ per SD = 2.23, 95% CI: [1.42, 3.51], *P* < 0.001; Table F in S3 Appendix). Test for Egger’s intercept suggested potential horizontal pleiotropy for the association between WC and HCC (intercept = −0.021, *P* = 0.039; Table G in S3 Appendix). Visual inspection of scatter plots and funnel plots revealed no apparent asymmetry or outliers (Figs A–Y in S6 Appendix). Steiger filtering confirmed the assumed causal direction for the included variants, and subsequent MR analyses yielded results consistent with the main findings (Tables B and C in S3 Appendix). Bidirectional MR analyses indicated potential bidirectional causal relationships between WC and glutamine, creatine, 2-hydroxybutyric acid, and hydroxyphenyllactic acid; WHR and hydroxyphenyllactic acid; and ABSI (women) and arachidonic acid (Table B in S3 Appendix). Nevertheless, the effect size for the reverse MR (metabolites → anthropometric measurements) were all weaker than the forward MR and distributed around 0. Notably, reverse MR analysis provided evidence suggesting that genetic liability to HCC was causally associated with higher circulating levels of GCDCA and TCDCA (Table C in S3 Appendix). The causal estimates were consistent across the primary IVW method and sensitivity analyses using other robust MR methods.

The results of multivariable MR suggested that the significant causal associations between anthropometric measurements and HCC persisted after adjusting for intermediate metabolites (Fig 7b and Table H in S3 Appendix). Regarding the causal associations between metabolites and HCC, 4 metabolites (creatine, arachidonic acid, 2-hydroxybutyric acid, and pyroglutamic acid) were significantly associated with HCC after adjusted for corresponding anthropometric measurements (Fig 7b). Integrating univariable MR and multivariable MR results, we estimated the mediated effect of metabolites using both the Difference and the Product method (Fig 7c). The Product method revealed a significant indirect effect of WHR on HCC, mediated through 2-hydroxybutyric acid (indirect effect = 0.09, 95% CI: [0.01, 0.17]) (Fig 7d). The difference method yielded directionally consistent but statistically non-significant estimates.

### Integration and summary of the results

**Fig 8:**
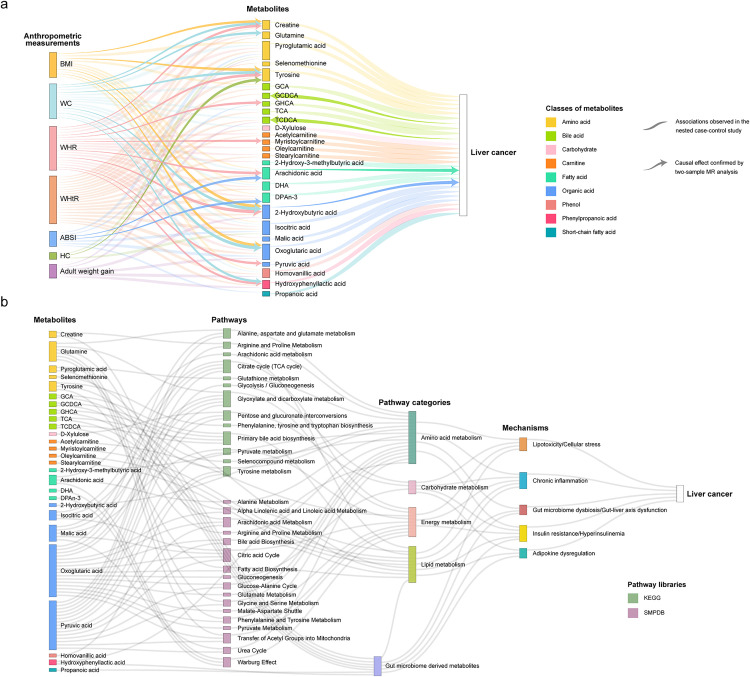
Integrated visualization linking anthropometric measurements, intermediate metabolites, enriched pathways, and established mechanistic hypotheses to liver cancer risk. **a** Sankey diagram mapping associations between seven anthropometric measurements (left), the 27 identified intermediate metabolites (center, colored by chemical class), and liver cancer risk (right). Flows and arrows represent identified links. The lighter flows indicate associations observed in the nested case-control study, while solid arrows denote potential causal relationships supported by MR analysis. **b** Diagram illustrating the connections of the 27 intermediate metabolites (left, colored by chemical class) to enriched biochemical pathways (colored by source database: KEGG or SMPDB), broader pathway categories, and established adiposity-liver cancer mechanistic hypotheses, ultimately linking to liver cancer. Gray flows indicate involvement or association between adjacent categories. BMI, body mass index; WC, waist circumference; WHR, waist-to-hip ratio; WHtR, waist-to-height ratio; ABSI, a body shape index; HC, hip circumference; KEGG, Kyoto Encyclopedia of Genes and Genomes; SMPDB, Small Molecule Pathway DataBase.

The results from the nested case-control study and the MR analysis are visually integrated in Fig 8a. Of the 108 associations initially identified in the nested case-control study, 23 were supported by the MR analysis, demonstrating statistical significance in both study designs and consistent effect directions. Notably, the vast majority of these confirmed associations (21 out of 23) were between anthropometric measurements and metabolites. Regarding potential causal links between metabolites and liver cancer, only the multivariable MR analysis adjusting for WHR indicated that arachidonic acid and 2-hydroxybutyric acid were positively associated with HCC risk. The multivariable MR analysis also provided evidence of a causal relationship of creatine (adjusted for WHR) and pyroglutamic acid (adjusted for HC) on HCC risk. However, the directions of these causal estimates were opposite to the ORs observed in the nested case-control study (Fig 3). Additionally, the reverse MR analysis identified significant causal effect of HCC on higher levels of TCDCA and GCDCA, suggesting that the alterations in these metabolites may occur after the onset of liver cancer.

We observed a notable difference between the extent of mediation suggested by observational analysis and that supported by MR. The observational mediation analysis (Fig 6) indicated that metabolic alterations might mediate a substantial proportion of the adiposity-liver cancer association. However, the MR analysis, which is less susceptible to confounding, provided causal evidence for a more specific subset of these mediators (Fig 7d). Fig 8b provides the biological context for the identified intermediate metabolites and enriched pathways, and integrates these findings into the established pathophysiological framework linking adiposity markers to liver cancer. The diagram sequentially connects the 27 intermediate metabolites to significantly enriched biochemical pathways (identified through pathway analysis), then maps these pathways to broader functional categories, and finally relates these connections to the five established mechanistic hypotheses linking adiposity to liver cancer. This visualization highlights the complex, interconnected metabolic link underlying the adiposity-liver cancer association, illustrating how observed alterations in core areas like energy, lipid, and amino acid metabolism align with established pathophysiological mechanisms linking adiposity to liver cancer.

## Discussion

Utilizing metabolomic profiling data from the prospective SMHS alongside two-sample MR analysis, this study systematically investigated potential metabolic mediators linking various anthropometric measurements to liver cancer risk. We identified 27 intermediate metabolites across diverse classes, forming five densely connected functional modules. Pathway analysis highlighted crucial alterations in amino acid, energy, and lipid metabolism, and MR analysis provided evidence supporting potential causal role for some of the observed associations.

To our knowledge, only one previous study by Assi and colleagues has examined the metabolic perturbations linking adiposity to liver cancer risk [58]. Based on a case-control study nested within the European Prospective Investigation into Cancer and Nutrition cohort (147 HCC cases and 147 matched controls), Assi and colleagues constructed a BMI-specific metabolic signature comprising 132 serum metabolites using partial least squares regression. They reported that this signature, primarily characterized by phosphatidylcholines (LysoPC aC18:2, LysoPC aC17:0, and PC aeC36:2) and tyrosine, fully mediated the association between BMI and HCC risk, with an estimated natural indirect effect of 1.56 (95% CI: [1.24, 1.96], *P* < 0.001). Consistent with their findings, our study also identified tyrosine as an important mediator that links multiple anthropometric measurements (BMI, WC, WHR, WHtR, and HC) to liver cancer risk. However, a key difference lies in the scope and approach: whereas Assi and colleagues focused on deriving a comprehensive BMI-related metabolic signature and evaluating its overall mediating effect, our study utilized seven anthropometric measurements to better characterize different types of fat accumulation, and concentrated on identifying individual intermediate metabolites, exploring relevant biochemical pathways, and providing evidence supporting potential causal relationships through MR analysis.

Our findings revealed complex dysregulations across amino acid, energy, and lipid metabolism, as well as perturbations in the gut-liver axis, all of which converge on established pathophysiological mechanisms linking adiposity to hepatocarcinogenesis, including insulin resistance [59], chronic inflammation [60], lipotoxicity [61], adipokine dysregulation [62], and gut microbiome dysbiosis [63]. Specifically, alterations in amino acid metabolism were prominent among the identified mediators, with five amino acids (creatine, glutamine, pyroglutamic acid, selenomethionine, and tyrosine) and several related pathways, such as Arginine and proline metabolism, Glutathione metabolism, Tyrosine metabolism, and Alanine, aspartate and glutamate metabolism, showing significant enrichment. Dysregulated tyrosine metabolism, reflected by altered tyrosine levels and enrichment of related pathways, is of particular interest. Tyrosine and other aromatic amino acids are associated with insulin resistance [64]. In addition, aromatic amino acids are closely intertwined with gut microbial activity, which can modulate host metabolic responses, potentially influencing systemic inflammation and metabolic health [65]. Our network analysis also positioned tyrosine as a key node connecting different metabolite modules, underscoring its potential integrative role. In addition to tyrosine’s role, the enrichment of Glutathione metabolism and Selenocompound metabolism pointed towards an imbalanced redox state and impaired antioxidant defense. Chronic inflammation and lipotoxicity, key mechanisms linking adiposity to liver cancer, are characterized by increased oxidative stress that can deplete glutathione and alter selenium homeostasis [66]. These findings suggest that adiposity may compromise the liver’s capacity to counteract oxidative damage, thereby promoting carcinogenesis.

Perturbations in central energy pathways were evident from intermediate metabolites like pyruvic acid, oxoglutaric acid, isocitric acid, and malic acid, and the enrichment of pathways such as the Citrate cycle (TCA cycle), Glycolysis/Gluconeogenesis, and Pyruvate metabolism. These alterations reflect dysregulated mitochondrial function and a potential shift in substrate utilization, closely linked to insulin resistance [67]. The enrichment of the Glucose-alanine cycle further highlighted impaired hepatic glucose metabolism and inter-organ crosstalk in the context of adiposity and insulin resistance. Network analysis showed a tight clustering of these energy-related metabolites (Module 3), supporting their coordinated dysregulation. Lipid metabolism was also significantly implicated. With regard to bile acid metabolism, several conjugated primary bile acids (GCA, GCDCA, TCA, TCDCA) were identified as intermediate metabolites. Adiposity is known to alter bile acid pool size and composition [10]. These changes impact signaling through nuclear (e.g., FXR) and membrane (e.g., TGR5) receptors, thereby influencing hepatic lipid and glucose metabolism, inflammation, and intestinal barrier integrity [68]. The clustering of these bile acids in our network analysis (Module 1) further emphasized their collective role. Apart from bile acids, several fatty acids (arachidonic acid, DHA, DPAn-3) and carnitines (acetylcarnitine, myristoylcarnitine, oleylcarnitine, stearylcarnitine) were also identified as mediators linking adiposity markers to liver cancer. Elevated arachidonic acid, a precursor for pro-inflammatory eicosanoids, directly points to increased chronic inflammation. Altered acylcarnitine profiles often signify impaired mitochondrial fatty acid β-oxidation, leading to accumulation of incompletely oxidized fatty acids and subsequent lipotoxicity and cellular stress [69]. Furthermore, the identification of propanoic acid and several bile acids as intermediate metabolites indicated that gut microbiome dysbiosis contributed to liver cancer risk by altering the profile of circulating microbial-derived or -modulated metabolites, which may then promote chronic inflammation, impair gut barrier function, and dysregulate host metabolism, thereby impacting all five mechanistic hypotheses linking adiposity to liver cancer. It is crucial to note that the identified metabolic alterations linking adiposity markers to liver cancer are not isolated but are highly interconnected. For instance, gut microbiome dysbiosis can influence bile acid profiles and systemic inflammation, which in turn can exacerbate insulin resistance and lipotoxicity in the liver [10,11]. The identified 27 intermediate metabolites likely represent key nodes within this complex network, reflecting a systemic metabolic perturbation that collectively drives the progression from adiposity to liver cancer.

Supporting these interconnected pathways, a parallel multiple mediation analysis provided substantial evidence for the mediating effects of metabolites on the associations between anthropometric measurements and liver cancer risk, as reflected by positive total IEs. Notably, negative IEs were observed for metabolite clusters including several fatty acids: DHA, DPAn-3, and arachidonic acid. These metabolites exhibited positive linear associations with anthropometric measurements and U-shaped non-linear associations with liver cancer risk. Given the inherent limitations of mediation analysis in capturing non-linear relationships, the interpretation of these negative IEs warrants caution. This observation underscores the complexity of the metabolic alterations involved in the development of liver cancer.

Given the high prevalence of HBV infection in our cancer cases (63.66%), disentangling the metabolic alterations of adiposity from those of chronic hepatitis is quite challenging. To distinguish these effects and ensure the robustness of our findings, we employed rigorous consistency-based screening and comprehensive statistical adjustment. While the primary identification of adiposity-associated metabolites was conducted in the total study population, we performed sensitivity analysis by excluding participants who were HBsAg-positive or had a history of chronic liver diseases to validate these associations. Only metabolites showing consistent associations were retained for further analysis. This stringent selection process confirms that the identified metabolic alterations correlate with adiposity markers are robust to HBV status. For the metabolite-liver cancer association, all models were adjusted for HBsAg status, self-reported history of chronic hepatitis, and cirrhosis. Similar sensitivity analysis was also conducted. Therefore, while HBV is a potent independent risk factor of liver cancer, our results strongly suggest that the metabolic signature identified here are primarily driven by adiposity.

Although we implemented sensitivity analyses to minimize potential impact of reverse causation, the inherent cross-sectional nature of these baseline measurements still fundamentally limits causal interpretation. Therefore, the supporting evidence from MR, which uses genetic variants as instruments less susceptible to confounding and reverse causation, significantly strengthens the potential causal interpretation of these associations. The MR analysis provided causal evidence for 23 associations identified in our observational analysis, which was particularly crucial for the associations between anthropometric measurements and metabolites. A significant causal indirect effect of WHR on liver cancer mediated through 2-hydroxybutyric acid were also suggested by the mediation MR analysis, which was consistent with the observational mediation analysis. The consistent identification of the 2-hydroxybutyric acid pathway across both methodologies underscores its potential role as a important metabolic link in adiposity-driven hepatocarcinogenesis.

A recent MR study by Ning and colleagues also investigated the causal effects of plasma metabolites on liver cancer risk [70]. While they identified 19 potential causal metabolites, there was no direct overlap with our study. This variation may be attributed to differences in the GWAS datasets utilized; our study leveraged more recent and larger GWAS summary statistics for both metabolites (CLSA) [48] and liver cancer (FinnGen) [42], offering enhanced statistical power. Nevertheless, both studies consistently suggest that only a small number of metabolites are causally associated with liver cancer. It is important to note that MR analysis could not be performed for all initially observed associations due to the unavailability of GWAS summary statistics for WHtR, adult weight gain, selenomethionine, and D-xylulose. However, among the associations that could be tested, less than one-third were confirmed by the MR analysis. Discrepancy was also observed between the extent of mediation suggested by observational regression models and that supported by MR analysis. Several factors might contribute to this discrepancy. First, the IVs for metabolites were based on GWAS with relatively small sample sizes (CLSA: ~8,000; FHS: 2,076), potentially limiting the statistical power to detect causal effects. Second, the standard two-sample MR methods employed are primarily designed to detect linear causal effects, potentially missing the complex, non-linear dose-response patterns (e.g., J-shaped curves) observed in our nested case-control study. Third, due to the lack of suitable GWAS datasets for metabolites in Asian populations, we utilized summary statistics derived from individuals of European ancestry. Similarly, the outcome GWAS data (from FinnGen) focused specifically on HCC, whereas our nested case-control study included liver cancer cases, encompassing HCC and other histological subtypes. Differences in population and outcome definition may affect the comparability and interpretation of the MR results and the cohort results. On the one hand, the consistency observed between the observational and MR findings for key metabolites (e.g., 2-hydroxybutyric acid) suggests that these associations likely represent fundamental, biologically conserved mechanisms linking adiposity to liver cancer, independent of ethnic background. On the other hand, null findings in the MR analysis for certain metabolites observed in the SMHS should not be definitively dismissed as non-causal. They may reflect population-specific effects driven by unique gene-environment interactions in Asian populations. Therefore, although MR offers valuable causal evidence, the results should be interpreted considering these potential limitations.

Beyond providing etiological insights, our findings have potential implications for liver cancer prevention strategies, particularly in the context of the rising global obesity epidemic. First, the identified metabolites could serve as candidate biomarkers for risk stratification. Integrating metabolic markers (such as 2-hydroxybutyric acid and specific bile acids) with traditional anthropometric measurement may be more efficient to identify high-risk populations. Individuals with both excess body fatness and altered metabolomic signatures might benefit from more intensive surveillance programs compared to those with ‘metabolically healthy obesity’. Second, these metabolites offer targets for monitoring metabolic interventions. While weight loss is the primary recommendation for obesity management, changes in body weight alone may not fully capture metabolic improvements. Monitoring the normalization of these specific intermediate metabolites could serve as a more sensitive measure for the efficacy of lifestyle or dietary interventions aimed at reducing liver cancer risk.

The current study has several strengths. First, the nested case-control study was conducted within a large-scale, population-based, and prospective cohort study, reducing susceptibility to selection bias and recall bias. Detailed information on covariates, such as HBsAg status, dietary habits, physical activity, lifestyle factors, and medical histories, allowed for comprehensive confounder control. Second, the current study integrated observational epidemiological data with MR analysis. This triangulation enhances causal inference, particularly for the cross-sectional associations between anthropometric measurements and metabolites. Finally, the targeted metabolomics approach utilized in the current study has higher sensitivity and provides absolute concentrations of metabolites (in μmol/L) compared to untargeted approaches. This further enabled the exploration of potential non-linear relationships, providing additional insights into the complex metabolic perturbations linking adiposity to liver cancer.

Despite the strengths, this study should be interpreted in consideration of several limitations. First, anthropometric measurements and metabolomic profiling were conducted only once at baseline giving the logistical challenges of large cohort studies. Single measurement might not fully capture long-term average exposure and could be subject to measurement error and intra-individual variability, potentially affecting the stability of the observed associations. Although the reproducibility of many metabolites over time has been reported [71,72], longitudinal studies with repeated measurements are needed to better account for within-person variations and to assess the dynamic changes of metabolites. Second, the meet-in-the-middle approach employed focused on identifying individual intermediate metabolites and could not account for intercorrelations or potential interactions among metabolites during the selection process. Therefore, some crucial metabolites with small effect sizes might have been missed. Third, based on MR sensitivity analyses, the core assumptions of MR may have been violated in some analyses. Considering these potential violations and other limitations discussed above, the MR results should be interpreted with caution. Fourth, due to the historical design of the SMHS, detailed histological subtype information for liver cancer cases was not available. In this case, our findings from the nested case-control study primarily reflect the metabolic alterations in the etiology of HCC (comprising 75%−85% of the cases) [1]. Finally, the participants in our nested case-control study were middle-aged Chinese men from an urban area, and the MR analysis primarily relied on GWAS summary statistics from populations of European ancestry. A notable limitation arises from the ancestry mismatch between our observational and the MR analysis. Genetic background, linkage disequilibrium patterns, and environmental exposures (e.g., diet habits and prevalence of viral hepatitis) differ significantly between these populations. This discrepancy warrants a cautious interpretation of our triangulation results. Future studies incorporating large-scale observational and genetic data from diverse ancestries are crucial to validate our findings and further explore other population-specific pathways linking adiposity to liver cancer.

In conclusion, this study offers metabolomic insights into the mechanisms linking adiposity markers to liver cancer risk by integrating prospective observational data with MR. We identified 27 potential intermediate metabolites and highlighted crucial alterations in pathways of energy, lipid, and amino acid metabolism. Our findings underscore the complexity of metabolic dysregulation involved in the adiposity-liver cancer association, enhance molecular understanding and identify potential metabolic targets for the primary prevention of liver cancer.

## Supporting information

S1 Checklist **Table A.** STROBE checklist. STrengthening the Reporting of OBservational studies in Epidemiology (STROBE) Statement—checklist of items that should be included in reports of observational studies, available at https://www.strobe-statement.org/, licensed under CC BY 4.0. Checklist available from https://www.strobe-statement.org/checklists/. **Table B.** STROBE-MR checklist. Checklist of recommended items to address in reports of mendelian randomization studies. Checklist available from https://www.strobe-mr.org/.
(XLSX)

S1 Text Supplementary methods. (DOCX)

S1 Appendix **Table A.** Definition of exposure variables. **Table B.** Distribution of anthropometric measurements. **Table C.** Spearman rank correlation coefficient. **Table D.** Associations between BMI and metabolite concentrations. **Table E.** Associations between WC and metabolite concentrations. **Table F.** Associations between WHR and metabolite concentrations. **Table G.** Associations between WHtR and metabolite concentrations. **Table H.** Associations between ABSI and metabolite concentrations. **Table I.** Associations between HC and metabolite concentrations. **Table J.** Associations between adult weight gain and metabolite concentrations. **Table K.** Associations between metabolite concentrations and liver cancer risk. **Table L.** Results of quantitative pathway analysis (KEGG database). **Table M.** Results of quantitative pathway analysis (SMPDB database). **Table N.** *P* values for partial correlations for the 27 intermediate metabolites (adjusted for age and fasting time, analysis conducted in controls only).
(XLSX)

S2 Appendix **Fig A.** Directed acyclic graphs for the association between anthropometric measurements and metabolites. **Fig B.** Directed acyclic graphs for the association between metabolites and liver cancer risk.
(DOCX)

S3 Appendix **Table A.** Information on GWAS summary datasets included in the MR analysis. **Table B.** Results of univariable MR (continuous outcomes). **Table C.** Results of univariable MR (categorical outcomes). **Table D.** Cochran’s *Q* tests for heterogeneity from IVW and MR-Egger methods. **Table E.** Results of MR-PRESSO global tests. **Table F.** Results of outlier-corrected MR analysis. **Table G.** MR-Egger intercept tests for horizontal pleiotropy. **Table H.** Results of multivariable MR.
(XLSX)

S4 Appendix **Fig A.** Enriched SMPDB pathways associated with adiposity markers and liver cancer risk, and their connection to intermediate metabolites. **Fig B.** KEGG pathways and relevant metabolites that linking adiposity markers to liver cancer.
(DOCX)

S5 Appendix **Fig A.** Hierarchical clustering results for intermediate metabolites in BMI-liver cancer association. **Fig B.** Hierarchical clustering results for intermediate metabolites in WC-liver cancer association. **Fig C.** Hierarchical clustering results for intermediate metabolites in WHR-liver cancer association. **Fig D.** Hierarchical clustering results for intermediate metabolites in WHtR-liver cancer association. **Fig E.** Hierarchical clustering results for intermediate metabolites in ABSI-liver cancer association. **Fig F.** Hierarchical clustering results for intermediate metabolites in HC-liver cancer association. **Fig G.** Hierarchical clustering results for intermediate metabolites in adult weight gain-liver cancer association.
(DOCX)

S6 Appendix **Fig A.** Scatter plot (a) and funnel plot (b) for the MR analysis between BMI and creatine. **Fig B.** Scatter plot (a) and funnel plot (b) for the MR analysis between BMI and tyrosine. **Fig C.** Scatter plot (a) and funnel plot (b) for the MR analysis between BMI and 2-hydroxybutyric acid. **Fig D.** Scatter plot (a) and funnel plot (b) for the MR analysis between BMI and oxoglutaric acid. **Fig E.** Scatter plot (a) and funnel plot (b) for the MR analysis between WC and glutamine. **Fig F.** Scatter plot (a) and funnel plot (b) for the MR analysis between WC and creatine. **Fig G.** Scatter plot (a) and funnel plot (b) for the MR analysis between WC and tyrosine. **Fig H.** Scatter plot (a) and funnel plot (b) for the MR analysis between WC and 2-hydroxybutyric acid. **Fig I.** Scatter plot (a) and funnel plot (b) for the MR analysis between WC and oxoglutaric acid. **Fig J.** Scatter plot (a) and funnel plot (b) for the MR analysis between WC and hydroxyphenyllactic acid. **Fig K.** Scatter plot (a) and funnel plot (b) for the MR analysis between WHR and creatine. **Fig L.** Scatter plot (a) and funnel plot (b) for the MR analysis between WHR and tyrosine. **Fig M.** Scatter plot (a) and funnel plot (b) for the MR analysis between WHR and GHCA. **Fig N.** Scatter plot (a) and funnel plot (b) for the MR analysis between WHR and myristoylcarnitine. **Fig O.** Scatter plot (a) and funnel plot (b) for the MR analysis between WHR and arachidonic acid. **Fig P.** Scatter plot (a) and funnel plot (b) for the MR analysis between WHR and 2-hydroxybutyric acid. **Fig Q.** Scatter plot (a) and funnel plot (b) for the MR analysis between WHR and pyruvic acid. **Fig R.** Scatter plot (a) and funnel plot (b) for the MR analysis between WHR and hydroxyphenyllactic acid. **Fig S.** Scatter plot (a) and funnel plot (b) for the MR analysis between ABSI_w and DPAn-3. **Fig T.** Scatter plot (a) and funnel plot (b) for the MR analysis between ABSI_w and arachidonic acid. **Fig U.** Scatter plot (a) and funnel plot (b) for the MR analysis between HC and tyrosine. **Fig V.** Scatter plot (a) and funnel plot (b) for the MR analysis between BMI and HCC. **Fig W.** Scatter plot (a) and funnel plot (b) for the MR analysis between WC and HCC. **Fig X.** Scatter plot (a) and funnel plot (b) for the MR analysis between WHR and HCC. **Fig Y.** Scatter plot (a) and funnel plot (b) for the MR analysis between ABSI_w and HCC.
(DOCX)

## Abbreviations

ABSI: a body shape index
BMI: body mass index
FDR: false discovery rate
GWAS: genome-wide association study
HBsAg: hepatitis B surface antigen
HBV: hepatitis B virus
HC: hip circumference
HCC: hepatocellular carcinoma
IE: indirect effect
IV: instrument variable
IVW: inverse-variance weighted
KEGG: Kyoto Encyclopedia of Genes and Genomes
LD: linkage disequilibrium
MR: mendelian randomization
OR: odds ratio
RCS: restricted cubic spline
SD: standard deviation
SMHS: Shanghai Men’s Health study
SMPDB: Small Molecule Pathway DataBase
SNP: single-nucleotide polymorphism
T2DM: type 2 diabetes mellitus
WC: waist circumference
WHR: waist-to-hip ratio
WHtR: waist-to-height ratio
