# Supplementary material for: Metabolomic insights into associations between adiposity markers and liver cancer risk: Results from a prospective cohort study and Mendelian randomization analysis
Source: PLoS Med. 2026 Feb 2;23(2):e1004910. doi: 10.1371/journal.pmed.1004910 (PMC12863527; doi:10.1371/journal.pmed.1004910)
Supplement: S1 Text — (DOCX) [file pmed.1004910.s002.docx]

**Metabolomic insights into associations between adiposity markers and liver cancer risk: results from a prospective cohort study and Mendelian randomization analysis**

Zhuo-Ying Li^1,2^, Hong-Lan Li^1,2^, Jing Wang^1,2^, Qiu-Ming Shen^1,2^, Yi-Xin Zou^1,2,3^, Dan-Ni Yang^1,2,4^, Yu-Ting Tan^1,2^, Yong-Bing Xiang^1,2,3,4,*^

**Affiliations:**

1. State Key Laboratory of System Medicine for Cancer, Shanghai Cancer Institute, Renji Hospital, Shanghai Jiao Tong University School of Medicine, Shanghai, China

2. Department of Epidemiology, Shanghai Cancer Institute, Shanghai, China

3. School of Public Health, Fudan University, Shanghai, China

4. School of Public Health, Shanghai Jiao Tong University School of Medicine, Shanghai, China

**ORCID:** Zhuo-Ying Li (0000-0003-4592-7136), Yong-Bing Xiang (0000-0002-3840-9915)

***** ybxiang@shsci.org

# S1 Text

# Supplementary Methods

1. **Methods of the nested case-control study**
   1. **Study design**

The present nested case-control study was conducted based on the Shanghai Men’s Health Study (SMHS), the rationale for which has been published elsewhere[1]. Briefly, the SMHS is a population-based and prospective cohort initiated in 2002-2006, including 61,469 Chinese men aged 40-74 years. Informed consent was obtained from each individual participant included in the cohort. Demographic characteristics, medical history, dietary habits, anthropometric measurements, physical activity participation, and other lifestyle factors of the study participants were collected at baseline interview. The food frequency questionnaire and physical activity questionnaire have been proven to have substantial reproducibility and validity[2,3]. Anthropometrics including height, weight, WC, and hip circumference (HC) of the cohort members were recorded according to a standardized protocol at the end of the baseline interview[1,4]. Two measurements were taken, with a tolerance for differences of less than 1.0 cm for height, WC, and HC, and 1.0 kg for weight. A third measurement was taken if the difference between the first two measurements exceeded the defined tolerances[4]. The average value of the two closest measurements was used for further analysis. Blood sample was collected from each of the 46,244 willing participants (75.1%) and were kept at 4℃, processed within 6 hours, and stored in -75℃ freezers[1]. Information on the date and time of sample collection, fasting status, and antibiotics use in the previous week was collected at the time of biospecimen collection. HBV infection status was determined by quantitating the hepatitis B surface antigen (HBsAg) levels and the details have been described in detail elsewhere[1,5].

To identify incident cancer cases in the SMHS, active follow-ups along with annual record linkage with the cancer registries and vital statistics registries were employed[1,6]. Up to now, three-rounds of follow-ups have been conducted, with high response rates (2004-2008: 97.6%; 2008-2011: 93.7% and 2012-2017: 93.6%). Possible cancer diagnoses were verified through home visits and review of medical records by clinical specialists and pathologists[1]. Liver cancer was defined based on the International Statistical Classification of Diseases, Injury and Causes of Death (9^th^ Revision) (ICD-9), including malignant neoplasm of liver and intrahepatic bile ducts (ICD-9 code 155).

By the end of 2016, 444 incident liver cancer cases were identified from the SMHS. The present nested case-control study was conducted based on cohort members who had adequate blood samples (including 326 liver cancer cases). The incidence density sampling method was employed to select matched controls for each liver cancer case. For each case, we randomly selected 1 control who were alive and free of cancer at the index time, matching in terms of age at blood collection (± 2 years), date of blood collection (± 30 days), time of blood collection (morning/afternoon), and antibiotics use during the preceding week (yes/no). After excluding 4 cases that lacked suitable controls, the nested case-control study comprised 322 liver cancer cases along with their respective controls[7].

- 1. **Quantitation of plasma metabolites**

The quantitation of plasma metabolites for 322 liver cancer cases and 322 controls was performed using a Q300 Kit from Metabo-Profile Biotechnology (Shanghai, China) Co., Ltd[8]. Sample preparation, instrument settings, and quality control were carried out according to the manufacturer’s protocol and have been described in detail elsewhere[7,8]. Briefly, an ultraperformance liquid chromatography coupled to tandem mass spectrometry (UPLC-MS/MS) system (ACQUITY UPLC-Xevo TQ-S, Waters Corp., Milford, MA, USA) was used to quantitate all targeted metabolites. A comprehensive set of rigorous quality control/assurance procedures was employed, including the use of test mixtures, pooled QC samples, reagent blanks, and calibrators, as detailed in previous studies[8]. Laboratory staff were blinded to the case-control status of the samples. Samples were analyzed in matched pairs to minimize batch effects. The raw data files generated by UPLC-MS/MS were processed using TMBQ software (v1.0, Human Metabolomics Institute, Shenzhen, Guangdong, China) for peak integration, calibration, and quantitation of each metabolite. We have comprehensively assessed the reproducibility and reliability of the metabolite quantitation in our previous publication and the results suggested that the quantitated results were robust and suitable for further analysis[7].

After standard quality control procedures, the absolute concentrations (μmol/L) of 186 known metabolites were quantified, including amino acids, benzenoids, benzoic acids, bile acids, carbohydrates, carnitines, fatty acids, indoles, nucleotides, organic acids, phenols, phenylpropanoic acids, pyridines, and short-chain fatty acids (SCFAs). Values below the lower limit of quantitation were imputed with half of the minimum observed concentration for each metabolite. Log_2_-transformation was applied to address the skewed distribution of metabolite concentrations.

- 1. **Selection of covariates**

Potential confounders were selected a priori based on their known associations with anthropometrics and liver cancer and their potential to affect plasma metabolites. We created directed acyclic graphs (DAGs) using the DAGitty web application to determine the minimal sufficient adjustment sets for the associations[9] (Supplementary Fig. S1, Supplementary Fig. S2). Based on the DAGs, the following covariates were included in the models examining anthropometrics-metabolites associations: age (years old, continuous), education level (elementary school or less, middle school college or above), personal income (< 1,000 Yuan/month, 1,000-2,999 Yuan/month, ≥ 3,000 Yuan/month), Chinese Food Pagoda (CHFP) score (0-45 points, continuous), cigarette smoking (never, ever), alcohol drinking (never, ever), and total physical activity (MET-hour/week, continuous). As for the models examining the metabolites-liver cancer associations, the minimal sufficient adjustment sets included age, CHFP score, cigarette smoking, alcohol drinking, BMI, physical activity, chronic hepatitis and cirrhosis (yes, no), medical history of cholelithiasis (yes, no), and medical history of T2DM (yes, no).

The CHFP score indicated adherence to the Chinese dietary guidelines and its calculation has been reported in our previous publications[10,11]. Considering the potential collinearity of seropositive HBsAg, medical history of chronic hepatitis, and medical history of cirrhosis, we combined these variables into one, i.e., chronic hepatitis and cirrhosis, to indicate potential liver damage at the time of blood collection.

- 1. **Identification of potential intermediate metabolites**

To identify metabolites associated with both anthropometric measurements and liver cancer risk, we utilized a two-step strategy. First, we used linear regression models to examine the associations between anthropometric measurements (explanatory variables) and plasma metabolites (dependent variables), adjusting for potential confounders mentioned above. Second, we fitted multivariable-adjusted conditional logistic regression models to examine the association between plasma metabolites and liver cancer risk. Next, we intersected the results from steps 1 and 2 to identify metabolites associated with both anthropometric measurements and liver cancer risk.

The anthropometric measurements and log_2_-transformed metabolite concentrations were scaled to Z-scores (mean=0, SD=1) before fitting the regression models to facilitate comparisons. The Benjamini-Hochberg false discovery rate (FDR) was applied to account for multiple comparisons across 186 metabolites[12]. Associations with an FDR less than 0.05 were retained. Restricted cubic spline (RCS) functions with three (10^th^, 50^th^, and 90^th^ percentile), four (5^th^, 35^th^, 65^th^, and 95^th^ percentile) and five knots (5^th^, 27.5^th^, 50^th^, 72.5^th^, and 95^th^ percentile) were fitted to both anthropometric measurements and metabolite concentrations to capture potential non-linear relationships[13,14]. The Akaike information criterion was used to select the best-fit models[15]. If the nonlinearity test for the RCS terms of an exposure variable was statistically significant (*P*_non-linear_ < 0.05), the association was retained for subsequent analyses.

To maximize statistical power to identify relevant metabolites, both liver cancer cases and controls were included in step 1. Nevertheless, to avoid the inclusion of associations entirely caused by reverse causation, we conducted three sensitivity analyses: repeating the analysis in control participants; repeating the analysis in participants with more than 2 years of follow-up; and repeating the analysis in participants without HBsAg seropositive or had a history of chronic hepatitis or cirrhosis. The observed anthropometrics-metabolites associations meeting the following criteria were excluded from further analyses: 1) for linear associations, β-values are in opposite directions, or 2) for non-linear associations, *P*_non-linear_>0.05. In the analysis of metabolites and liver cancer, to rule out the potential residual confounding of viral hepatitis, we performed sensitivity analyses by excluding participants who were HBsAg seropositive or had a history of chronic hepatitis or cirrhosis. Unconditional logistic regression was used as the matched pairs have been split.

1. **Methods of the two‑sample Mendelian randomization**
   1. **Data source**

Genome-wide association study (GWAS) summary statistics from non-overlapping samples for anthropometric measurements, metabolites, and liver cancer were obtained, as detailed in Supplementary Table S2. Briefly, the summary statistics for anthropometric measurements were obtained from the Genetic Investigation of ANthropometric Traits (GIANT) consortium and the UK Biobank (UKB)[16–20]. Genetic variants associated with metabolites were derived from the Canadian Longitudinal Study on Aging (CLSA) cohort and the Framingham Heart Study (FHS)[21–23]. Summary statistics for the outcome were obtained from the FinnGen study (R11 data release; 609 hepatocellular carcinoma (HCC) cases and 345,118 controls)[24]. All included GWAS were conducted on individuals of European descent. We did not find available GWAS summary data for WHtR, adult weight gain, selenomethionine and D-xylulose. Therefore, these traits were not included in the MR analysis.

- 1. **Selection of instrument variables and data harmonization**

The selection of instrument variable (IV) was guided by three core assumptions of MR: (1) Relevance: the IV is strongly associated with the exposure; (2) Independence: the IV is not associated with the outcome via a confounding pathway; (3) Exclusion restriction: the IV does not affect the outcome directly, only possibly indirectly via the exposure[25,26].

For anthropometric measurements and HCC (in bidirectional MR), all single nucleotide polymorphisms (SNPs) associated with the exposure at a genome-wide significance threshold (*P* < 5×10^-8^) were selected as IVs. For metabolites, a less stringent threshold (*P* < 5×10^-6^) was used due to relatively small sample sizes of the CLSA (~8,000) and FHS (2,076) GWAS datasets. Using the PLINK clumping method, the resulting SNPs were pruned with a linkage disequilibrium (LD) threshold of r^2^ < 0.001 within a 10,000-kilobase (kb) window to exclude SNPs unlikely to be independently associated with the phenotype. Where an instrument SNP was not available in the outcome dataset, we searched for LD proxies (r^2^ > 0.6) for the target SNP[27]. When multiple proxy SNPs existed, we extracted the one with the highest r^2^ with the target SNP. The European super-population from the 1,000 Genomes Project reference dataset, which included only bi-allelic SNPs with a minor allele frequency (MAF) > 0.01, was used as the LD reference panel.

The exposure and outcome datasets were harmonized to ensure that the effect of each SNP corresponded to the same allele. Allele frequency information was used to infer forward strand alleles. Palindromic SNPs with a minor allele frequency above 0.42 were discarded from the analysis[28].

- 1. **Univariable Mendelian randomization analysis and sensitivity analyses**

The primary univariable Mendelian randomization (UVMR) analysis was conducted using the multiplicative random-effects inverse-variance weighted (IVW) method, as it provides the most precise estimates under the assumption that all SNPs are valid IVs[29]. To assess the robustness of the main findings, the MR-Egger[30], weighted median[31], and weighted mode[32] methods were additionally applied, as they produce valid causal estimates under different assumptions. When only one SNP remained as a valid IV, the Wald ratio method was used to calculate the causal estimates[26].

In addition to employing robust MR methods, various sensitivity analyses were performed to assess potential violations of MR assumptions and to validate the causal associations identified by the IVW method. Cochran’s Q statistic was used to evaluate heterogeneity among variant-specific causal estimates[26,33]. The MR-Egger intercept test was used to assess horizontal pleiotropy, with an intercept term differing from zero indicating the presence of directional pleiotropy[30]. The Mendelian Randomization Pleiotropy RESidual Sum and Outlier (MR-PRESSO) global test, outlier test, and distortion test were conducted to detect and remove horizontal pleiotropic variants[34]. If the global test suggested significant horizontal pleiotropy for an association, we repeated the MR analysis after excluding the identified pleiotropic variants. In addition, scatter plots and funnel plots were generated to visually evaluate the precision of the variant-specific causal estimates and the presence of directional pleiotropy effect. To reduce the risk of reverse causality in MR analyses, we applied Steiger filtering to remove variants from the analysis if a given SNP explained more variance in the exposure than the outcome[35]. Additionally, bidirectional MR was conducted to clarify the direction of associations between the exposure and the outcome.

- 1. **Multivariable Mendelian randomization and mediation analysis**

To further investigate the independent causal effects of adiposity and metabolites on the risk of liver cancer, and to investigate the potential mediating role of metabolites in the association between adiposity and liver cancer risk, we performed multivariable Mendelian randomization (MVMR) analysis[36]. In MVMR, SNPs associated with both anthropometric measurements and metabolites are allowed to be included as IVs in the analysis[37–39].

The indirect (mediated) effect through metabolites was then examined using two approaches: the Difference method and the Product method (Fig. 1)[37]. In the Difference method, the indirect effect was calculated as the difference between the total effect (from UVMR) and the direct effect (from MVMR). In the Product method, the indirect effect was calculated as the product of the causal effect of the exposure on the mediator (from UVMR) and the causal effect of the mediator on the outcome, adjusted for the exposure (from MVMR). The mediation proportion was calculated as the ratio of the indirect effect to the total effect.

**Reference**

1. Shu XO, Li H, Yang G, Gao J, Cai H, Takata Y, et al. Cohort profile: the Shanghai Men’s Health Study. Int J Epidemiol. 2015;44: 810–818. doi:10.1093/ije/dyv013

2. Jurj AL, Wen W, Xiang YB, Matthews CE, Liu D, Zheng W, et al. Reproducibility and validity of the Shanghai Men’s Health Study physical activity questionnaire. Am J Epidemiol. 2007;165: 1124–1133. doi:10.1093/aje/kwk119

3. Villegas R, Yang G, Liu D, Xiang YB, Cai H, Zheng W, et al. Validity and reproducibility of the food-frequency questionnaire used in the Shanghai Men’s Health Study. Br J Nutr. 2007;97: 993–1000. doi:10.1017/S0007114507669189

4. Li ZY, Tan YT, Wang J, Fang J, Liu DK, Li HL, et al. Dose-response relationship between fat distribution and liver cancer incidence: a prospective cohort study in Chinese men. Cancer Epidemiol. 2022;76: 102091. doi:10.1016/j.canep.2021.102091

5. Yang Y, Gao J, Li HL, Zheng W, Yang G, Zhang W, et al. Dose-response association between hepatitis B surface antigen levels and liver cancer risk in Chinese men and women. Int J Cancer. 2016;139: 355–362. doi:10.1002/ijc.30086

6. Rao C, Yang G, Hu J, Ma J, Xia W, Lopez AD. Validation of cause-of-death statistics in urban China. Int J Epidemiol. 2007;36: 642–651. doi:10.1093/ije/dym003

7. Li ZY, Shen QM, Wang J, Tuo JY, Tan YT, Li HL, et al. Prediagnostic plasma metabolite concentrations and liver cancer risk: a population-based study of Chinese men. eBioMedicine. 2024;100: 104990. doi:10.1016/j.ebiom.2024.104990

8. Xie G, Wang L, Chen T, Zhou K, Zhang Z, Li J, et al. A metabolite array technology for precision medicine. Anal Chem. 2021;93: 5709–5717. doi:10.1021/acs.analchem.0c04686

9. Textor J, van der Zander B, Gilthorpe MS, Liskiewicz M, Ellison GT. Robust causal inference using directed acyclic graphs: the R package “dagitty.” Int J Epidemiol. 2016;45: 1887–1894. doi:10.1093/ije/dyw341

10. Nguyen S, Li H, Yu D, Gao J, Gao Y, Tran H, et al. Adherence to dietary recommendations and colorectal cancer risk: results from two prospective cohort studies. Int J Epidemiol. 2020;49: 270–280. doi:10.1093/ije/dyz118

11. Yu DX, Zhang XL, Xiang YB, Yang G, Li HL, Gao YT, et al. Adherence to dietary guidelines and mortality: a report from prospective cohort studies of 134,000 Chinese adults in urban Shanghai. Am J Clin Nutr. 2014;100: 693–700. doi:10.3945/ajcn.113.079194

12. Benjamini Y, Hochberg Y. Controlling the false discovery rate: a practical and powerful approach to multiple testing. J R Stat Soc Ser B Methodol. 1995;57: 289–300. doi:10.1111/j.2517-6161.1995.tb02031.x

13. Durrleman S, Simon R. Flexible regression models with cubic splines. Stat Med. 1989;8: 551–561. doi:10.1002/sim.4780080504

14. Desquilbet L, Mariotti F. Dose‐response analyses using restricted cubic spline functions in public health research. Stat Med. 2010;29: 1037–1057. doi:10.1002/sim.3841

15. Harrell FE. Regression modeling strategies: with applications to linear models, logistic and ordinal regression, and survival analysis. Springer; 2015. Available: https://link.springer.com/10.1007/978-3-319-19425-7

16. Yengo L, Sidorenko J, Kemper KE, Zheng Z, Wood AR, Weedon MN, et al. Meta-analysis of genome-wide association studies for height and body mass index in ∼700000 individuals of European ancestry. Hum Mol Genet. 2018;27: 3641–3649. doi:10.1093/hmg/ddy271

17. Bycroft C, Freeman C, Petkova D, Band G, Elliott LT, Sharp K, et al. The UK Biobank resource with deep phenotyping and genomic data. Nature. 2018;562: 203–209. doi:10.1038/s41586-018-0579-z

18. Pulit SL, Stoneman C, Morris AP, Wood AR, Glastonbury CA, Tyrrell J, et al. Meta-analysis of genome-wide association studies for body fat distribution in 694 649 individuals of European ancestry. Hum Mol Genet. 2019;28: 166–174. doi:10.1093/hmg/ddy327

19. Ruth Mitchell E. MRC IEU UK Biobank GWAS pipeline version 2. In: data.bris [Internet]. 20 Feb 2019 [cited 14 Oct 2024]. doi:10.5523/bris.pnoat8cxo0u52p6ynfaekeigi

20. Christakoudi S, Evangelou E, Riboli E, Tsilidis KK. GWAS of allometric body-shape indices in UK Biobank identifies loci suggesting associations with morphogenesis, organogenesis, adrenal cell renewal and cancer. Sci Rep. 2021;11: 10688. doi:10.1038/s41598-021-89176-6

21. Chen Y, Lu T, Pettersson-Kymmer U, Stewart ID, Butler-Laporte G, Nakanishi T, et al. Genomic atlas of the plasma metabolome prioritizes metabolites implicated in human diseases. Nat Genet. 2023;55: 44–53. doi:10.1038/s41588-022-01270-1

22. Raina P, Wolfson C, Kirkland S, Griffith LE, Balion C, Cossette B, et al. Cohort profile: the Canadian longitudinal study on aging (CLSA). Int J Epidemiol. 2019;48: 1752–1753j.

23. Rhee EP, Ho JE, Chen M-H, Shen D, Cheng S, Larson MG, et al. A genome-wide association study of the human metabolome in a community-based cohort. Cell Metab. 2013;18: 130–143. doi:10.1016/j.cmet.2013.06.013

24. Kurki MI, Karjalainen J, Palta P, Sipilä TP, Kristiansson K, Donner KM, et al. FinnGen provides genetic insights from a well-phenotyped isolated population. Nature. 2023;613: 508–518. doi:10.1038/s41586-022-05473-8

25. Sekula P, Del Greco M F, Pattaro C, Köttgen A. Mendelian Randomization as an Approach to Assess Causality Using Observational Data. J Am Soc Nephrol. 2016;27: 3253–3265. doi:10.1681/ASN.2016010098

26. Burgess S, Thompson SG. Mendelian randomization: methods for causal inference using genetic variants. Second edition. Boca Raton London New York: CRC Press; 2021.

27. Lyon M, Andrews SJ, Elsworth B, Gaunt TR, Hemani G, Marcora E. The variant call format provides efficient and robust storage of GWAS summary statistics. bioRxiv; 2020. p. 2020.05.29.115824. doi:10.1101/2020.05.29.115824

28. Hemani G, Bowden J, Davey Smith G. Evaluating the potential role of pleiotropy in Mendelian randomization studies. Hum Mol Genet. 2018;27: R195–R208. doi:10.1093/hmg/ddy163

29. Burgess S, Butterworth A, Thompson SG. Mendelian randomization analysis with multiple genetic variants using summarized data. Genet Epidemiol. 2013;37: 658–665. doi:10.1002/gepi.21758

30. Bowden J, Davey Smith G, Burgess S. Mendelian randomization with invalid instruments: effect estimation and bias detection through Egger regression. Int J Epidemiol. 2015;44: 512–525. doi:10.1093/ije/dyv080

31. Bowden J, Davey Smith G, Haycock PC, Burgess S. Consistent Estimation in Mendelian Randomization with Some Invalid Instruments Using a Weighted Median Estimator. Genet Epidemiol. 2016;40: 304–314. doi:10.1002/gepi.21965

32. Hartwig FP, Davey Smith G, Bowden J. Robust inference in summary data Mendelian randomization via the zero modal pleiotropy assumption. Int J Epidemiol. 2017;46: 1985–1998. doi:10.1093/ije/dyx102

33. Greco M FD, Minelli C, Sheehan NA, Thompson JR. Detecting pleiotropy in Mendelian randomisation studies with summary data and a continuous outcome. Stat Med. 2015;34: 2926–2940. doi:10.1002/sim.6522

34. Verbanck M, Chen C-Y, Neale B, Do R. Detection of widespread horizontal pleiotropy in causal relationships inferred from Mendelian randomization between complex traits and diseases. Nat Genet. 2018;50: 693–698. doi:10.1038/s41588-018-0099-7

35. Hemani G, Tilling K, Davey Smith G. Orienting the causal relationship between imprecisely measured traits using GWAS summary data. PLoS Genet. 2017;13: e1007081. doi:10.1371/journal.pgen.1007081

36. Burgess S, Thompson SG. Multivariable Mendelian randomization: the use of pleiotropic genetic variants to estimate causal effects. Am J Epidemiol. 2015;181: 251–260. doi:10.1093/aje/kwu283

37. Carter AR, Sanderson E, Hammerton G, Richmond RC, Davey Smith G, Heron J, et al. Mendelian randomisation for mediation analysis: current methods and challenges for implementation. Eur J Epidemiol. 2021;36: 465–478. doi:10.1007/s10654-021-00757-1

38. Burgess S, Daniel RM, Butterworth AS, Thompson SG, EPIC-InterAct Consortium. Network Mendelian randomization: using genetic variants as instrumental variables to investigate mediation in causal pathways. Int J Epidemiol. 2015;44: 484–495. doi:10.1093/ije/dyu176

39. Sanderson E. Multivariable Mendelian randomization and mediation. Cold Spring Harb Perspect Med. 2021;11: a038984. doi:10.1101/cshperspect.a038984
