# Supplementary material for: Metabolomic insights into associations between adiposity markers and liver cancer risk: Results from a prospective cohort study and Mendelian randomization analysis
Source: PLoS Med. 2026 Feb 2;23(2):e1004910. doi: 10.1371/journal.pmed.1004910 (PMC12863527; doi:10.1371/journal.pmed.1004910)
Supplement: S2 Appendix — Fig A. Directed acyclic graphs for the association between anthropometric measurements and metabolites. Fig B. Directed acyclic graphs for the association between metabolites and liver cancer risk. (DOCX) [file pmed.1004910.s004.docx]

**Metabolomic insights into associations between adiposity markers and liver cancer risk: results from a prospective cohort study and Mendelian randomization analysis**

Zhuo-Ying Li^1,2^, Hong-Lan Li^1,2^, Jing Wang^1,2^, Qiu-Ming Shen^1,2^, Yi-Xin Zou^1,2,3^, Dan-Ni Yang^1,2,4^, Yu-Ting Tan^1,2^, Yong-Bing Xiang^1,2,3,4,*^

**Affiliations:**

1. State Key Laboratory of System Medicine for Cancer, Shanghai Cancer Institute, Renji Hospital, Shanghai Jiao Tong University School of Medicine, Shanghai, China

2. Department of Epidemiology, Shanghai Cancer Institute, Shanghai, China

3. School of Public Health, Fudan University, Shanghai, China

4. School of Public Health, Shanghai Jiao Tong University School of Medicine, Shanghai, China

**ORCID:** Zhuo-Ying Li (0000-0003-4592-7136), Yong-Bing Xiang (0000-0002-3840-9915)

***** ybxiang@shsci.org

**S2 Appendix**

[Fig A. Directed acyclic graphs for the association between anthropometric measurements and metabolites. 3](#_Toc214370371)

[Fig B. Directed acyclic graphs for the association between metabolites and liver cancer risk. 4](#_Toc214370372)





# Fig A. Directed acyclic graphs for the association between anthropometric measurements and metabolites.





# Fig B. Directed acyclic graphs for the association between metabolites and liver cancer risk.
