# Supplementary material for: Metabolomic insights into associations between adiposity markers and liver cancer risk: Results from a prospective cohort study and Mendelian randomization analysis
Source: PLoS Med. 2026 Feb 2;23(2):e1004910. doi: 10.1371/journal.pmed.1004910 (PMC12863527; doi:10.1371/journal.pmed.1004910)
Supplement: S5 Appendix — Fig A. Hierarchical clustering results for intermediate metabolites in BMI-liver cancer association. Fig B. Hierarchical clustering results for intermediate metabolites in WC-liver cancer association. Fig C. Hierarchical clustering results for intermediate metabolites in WHR-liver cancer association. Fig D. Hierarchical clustering results for intermediate metabolites in WHtR-liver cancer association. Fig E. Hierarchical clustering results for intermediate metabolites in ABSI-liver cancer association. Fig F. Hierarchical clustering results for intermediate metabolites in HC-liver cancer association. Fig G. Hierarchical clustering results for intermediate metabolites in adult weight gain-liver cancer association. (DOCX) [file pmed.1004910.s007.docx]

**Metabolomic insights into associations between adiposity markers and liver cancer risk: results from a prospective cohort study and Mendelian randomization analysis**

Zhuo-Ying Li^1,2^, Hong-Lan Li^1,2^, Jing Wang^1,2^, Qiu-Ming Shen^1,2^, Yi-Xin Zou^1,2,3^, Dan-Ni Yang^1,2,4^, Yu-Ting Tan^1,2^, Yong-Bing Xiang^1,2,3,4,*^

**Affiliations:**

1. State Key Laboratory of System Medicine for Cancer, Shanghai Cancer Institute, Renji Hospital, Shanghai Jiao Tong University School of Medicine, Shanghai, China

2. Department of Epidemiology, Shanghai Cancer Institute, Shanghai, China

3. School of Public Health, Fudan University, Shanghai, China

4. School of Public Health, Shanghai Jiao Tong University School of Medicine, Shanghai, China

**ORCID:** Zhuo-Ying Li (0000-0003-4592-7136), Yong-Bing Xiang (0000-0002-3840-9915)

***** ybxiang@shsci.org

**S5 Appendix**

[Fig A. Hierarchical clustering results for intermediate metabolites in BMI-liver cancer association. 3](#_Toc214390273)

[Fig B. Hierarchical clustering results for intermediate metabolites in WC-liver cancer association. 4](#_Toc214390274)

[Fig C. Hierarchical clustering results for intermediate metabolites in WHR-liver cancer association. 5](#_Toc214390275)

[Fig D. Hierarchical clustering results for intermediate metabolites in WHtR-liver cancer association. 6](#_Toc214390276)

[Fig E. Hierarchical clustering results for intermediate metabolites in ABSI-liver cancer association. 7](#_Toc214390277)

[Fig F. Hierarchical clustering results for intermediate metabolites in HC-liver cancer association. 8](#_Toc214390278)

[Fig G. Hierarchical clustering results for intermediate metabolites in adult weight gain-liver cancer association. 9](#_Toc214390279)


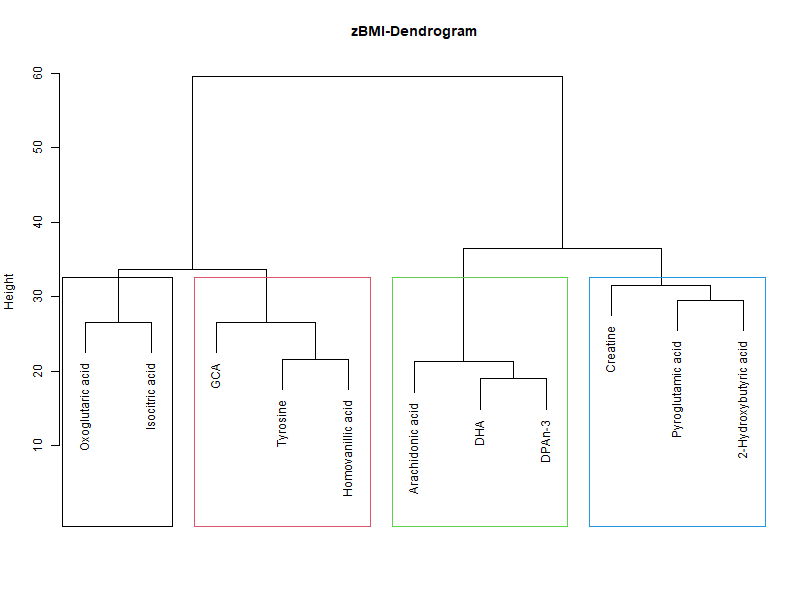


# Fig A. Hierarchical clustering results for intermediate metabolites in BMI-liver cancer association.


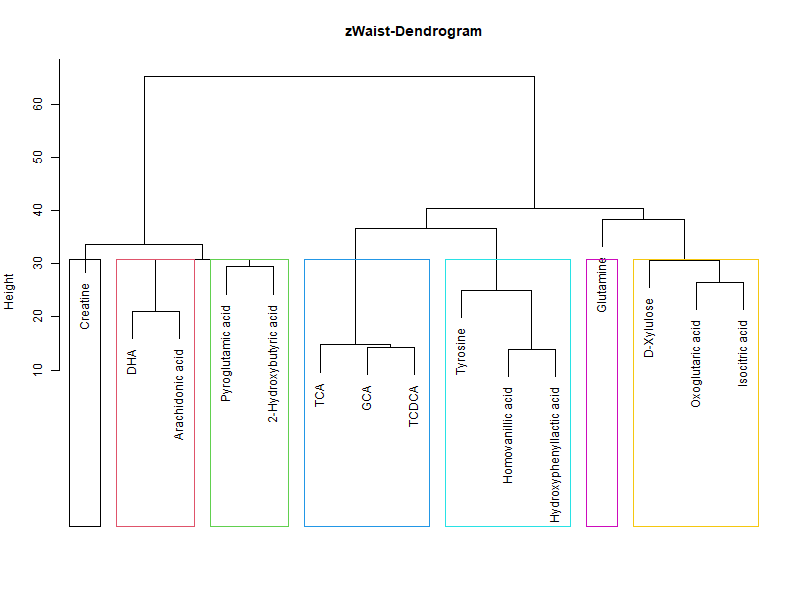


# Fig B. Hierarchical clustering results for intermediate metabolites in WC-liver cancer association.


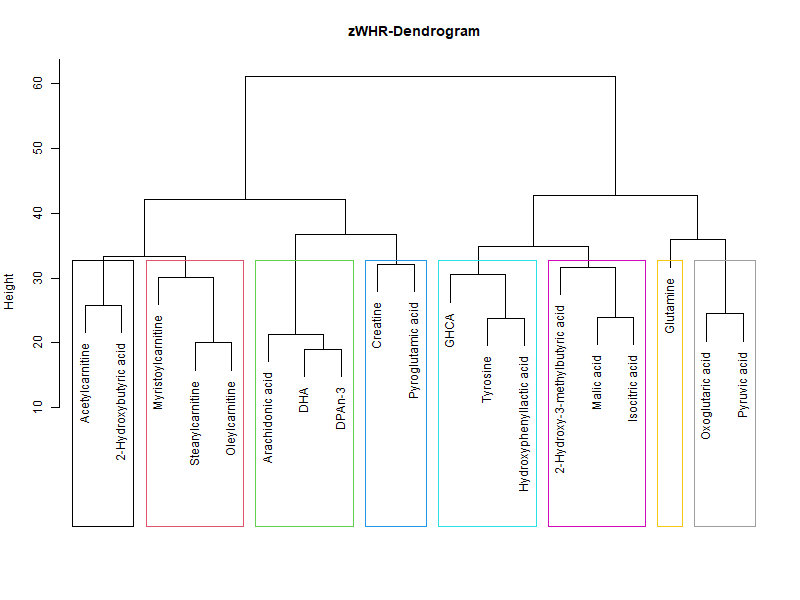


# Fig C. Hierarchical clustering results for intermediate metabolites in WHR-liver cancer association.


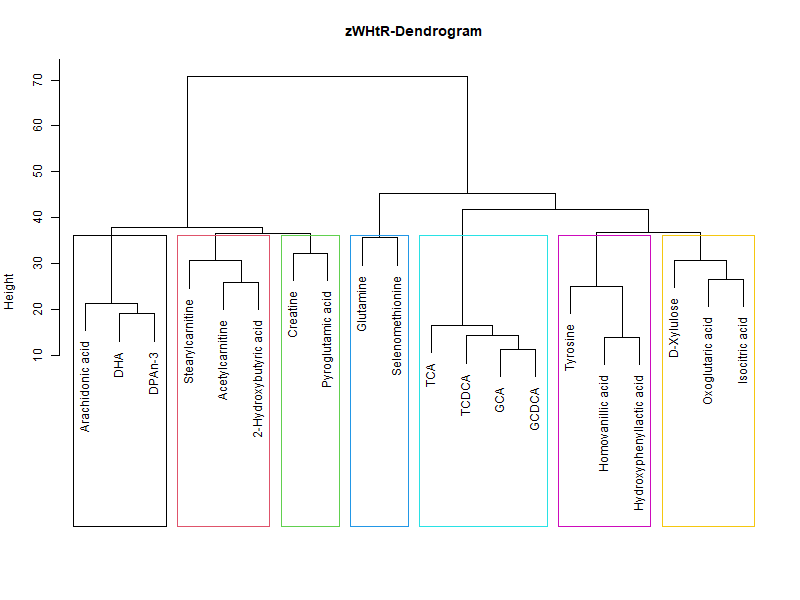


# Fig D. Hierarchical clustering results for intermediate metabolites in WHtR-liver cancer association.


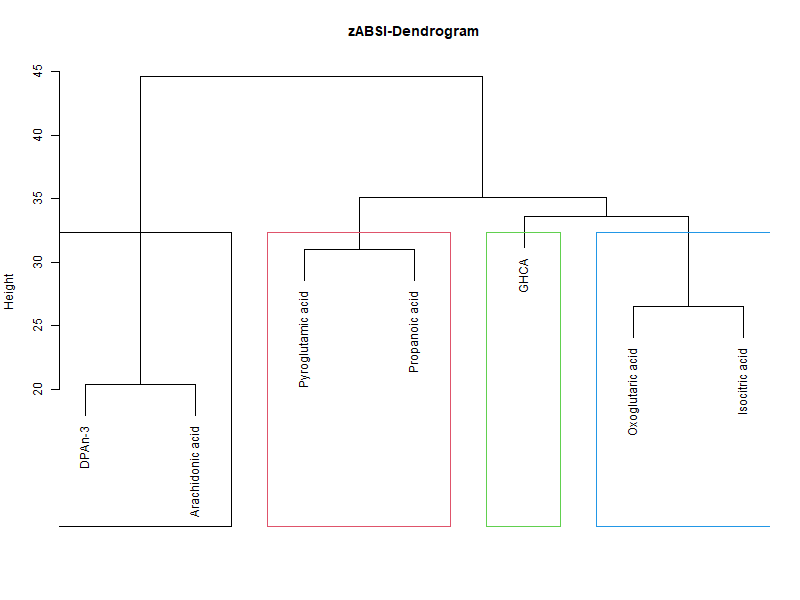


# Fig E. Hierarchical clustering results for intermediate metabolites in ABSI-liver cancer association.


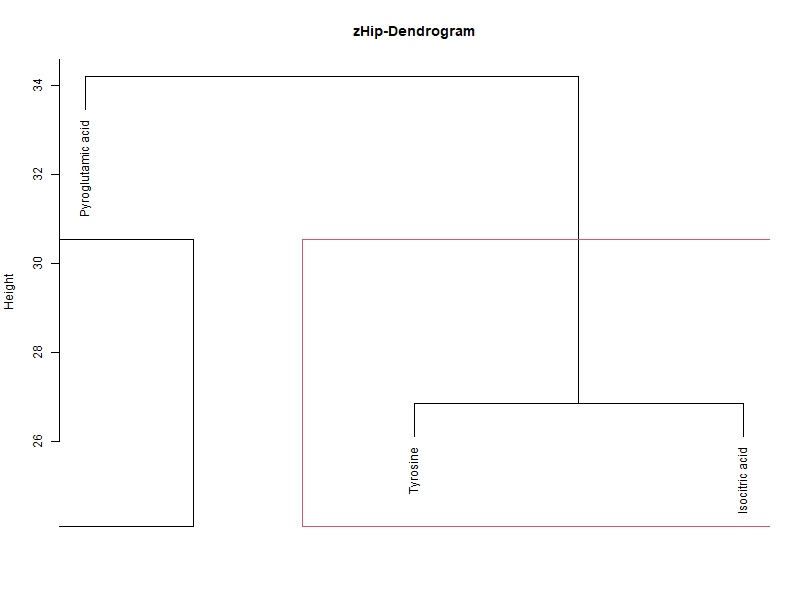


# Fig F. Hierarchical clustering results for intermediate metabolites in HC-liver cancer association.


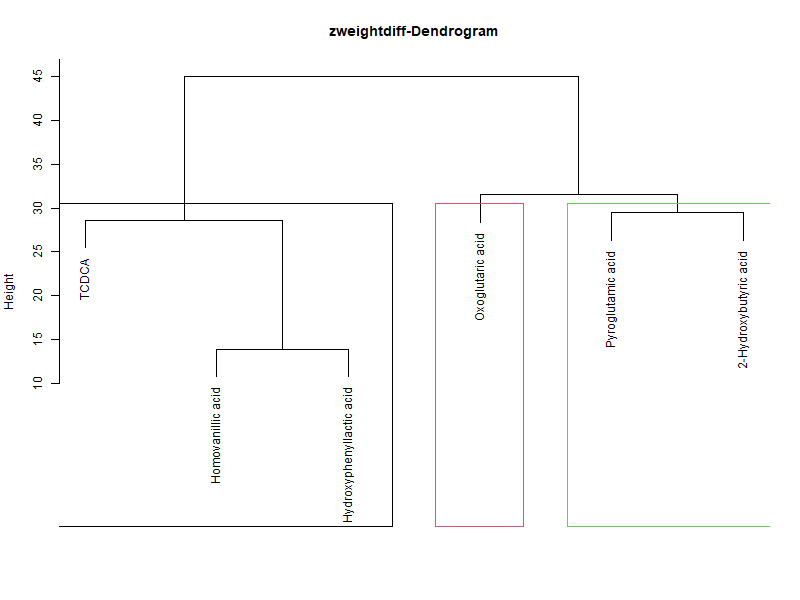


# Fig G. Hierarchical clustering results for intermediate metabolites in adult weight gain-liver cancer association.
