# Supplementary material for: Metabolomic insights into associations between adiposity markers and liver cancer risk: Results from a prospective cohort study and Mendelian randomization analysis
Source: PLoS Med. 2026 Feb 2;23(2):e1004910. doi: 10.1371/journal.pmed.1004910 (PMC12863527; doi:10.1371/journal.pmed.1004910)
Supplement: S6 Appendix — Fig A. Scatter plot (a) and funnel plot (b) for the MR analysis between BMI and creatine. Fig B. Scatter plot (a) and funnel plot (b) for the MR analysis between BMI and tyrosine. Fig C. Scatter plot (a) and funnel plot (b) for the MR analysis between BMI and 2-hydroxybutyric acid. Fig D. Scatter plot (a) and funnel plot (b) for the MR analysis between BMI and oxoglutaric acid. Fig E. Scatter plot (a) and funnel plot (b) for the MR analysis between WC and glutamine. Fig F. Scatter plot (a) and funnel plot (b) for the MR analysis between WC and creatine. Fig G. Scatter plot (a) and funnel plot (b) for the MR analysis between WC and tyrosine. Fig H. Scatter plot (a) and funnel plot (b) for the MR analysis between WC and 2-hydroxybutyric acid. Fig I. Scatter plot (a) and funnel plot (b) for the MR analysis between WC and oxoglutaric acid. Fig J. Scatter plot (a) and funnel plot (b) for the MR analysis between WC and hydroxyphenyllactic acid. Fig K. Scatter plot (a) and funnel plot (b) for the MR analysis between WHR and creatine. Fig L. Scatter plot (a) and funnel plot (b) for the MR analysis between WHR and tyrosine. Fig M. Scatter plot (a) and funnel plot (b) for the MR analysis between WHR and GHCA. Fig N. Scatter plot (a) and funnel plot (b) for the MR analysis between WHR and myristoylcarnitine. Fig O. Scatter plot (a) and funnel plot (b) for the MR analysis between WHR and arachidonic acid. Fig P. Scatter plot (a) and funnel plot (b) for the MR analysis between WHR and 2-hydroxybutyric acid. Fig Q. Scatter plot (a) and funnel plot (b) for the MR analysis between WHR and pyruvic acid. Fig R. Scatter plot (a) and funnel plot (b) for the MR analysis between WHR and hydroxyphenyllactic acid. Fig S. Scatter plot (a) and funnel plot (b) for the MR analysis between ABSI_w and DPAn-3. Fig T. Scatter plot (a) and funnel plot (b) for the MR analysis between ABSI_w and arachidonic acid. Fig U. Scatter plot (a) and funnel plot (b) for the MR analysis between HC and tyrosine. [file pmed.1004910.s008.docx]

**Metabolomic insights into associations between adiposity markers and liver cancer risk: results from a prospective cohort study and Mendelian randomization analysis**

Zhuo-Ying Li^1,2^, Hong-Lan Li^1,2^, Jing Wang^1,2^, Qiu-Ming Shen^1,2^, Yi-Xin Zou^1,2,3^, Dan-Ni Yang^1,2,4^, Yu-Ting Tan^1,2^, Yong-Bing Xiang^1,2,3,4,*^

**Affiliations:**

1. State Key Laboratory of System Medicine for Cancer, Shanghai Cancer Institute, Renji Hospital, Shanghai Jiao Tong University School of Medicine, Shanghai, China

2. Department of Epidemiology, Shanghai Cancer Institute, Shanghai, China

3. School of Public Health, Fudan University, Shanghai, China

4. School of Public Health, Shanghai Jiao Tong University School of Medicine, Shanghai, China

**ORCID:** Zhuo-Ying Li (0000-0003-4592-7136), Yong-Bing Xiang (0000-0002-3840-9915)

***** ybxiang@shsci.org

**S6 Appendix**

[Fig A. Scatter plot (a) and funnel plot (b) for the MR analysis between BMI and creatine. 3](#_Toc214390083)

[Fig B. Scatter plot (a) and funnel plot (b) for the MR analysis between BMI and tyrosine. 4](#_Toc214390084)

[Fig C. Scatter plot (a) and funnel plot (b) for the MR analysis between BMI and 2-hydroxybutyric acid. 5](#_Toc214390085)

[Fig D. Scatter plot (a) and funnel plot (b) for the MR analysis between BMI and oxoglutaric acid. 6](#_Toc214390086)

[Fig E. Scatter plot (a) and funnel plot (b) for the MR analysis between WC and glutamine. 7](#_Toc214390087)

[Fig F. Scatter plot (a) and funnel plot (b) for the MR analysis between WC and creatine. 8](#_Toc214390088)

[Fig G. Scatter plot (a) and funnel plot (b) for the MR analysis between WC and tyrosine. 9](#_Toc214390089)

[Fig H. Scatter plot (a) and funnel plot (b) for the MR analysis between WC and 2-hydroxybutyric acid. 10](#_Toc214390090)

[Fig I. Scatter plot (a) and funnel plot (b) for the MR analysis between WC and oxoglutaric acid. 11](#_Toc214390091)

[Fig J. Scatter plot (a) and funnel plot (b) for the MR analysis between WC and hydroxyphenyllactic acid. 12](#_Toc214390092)

[Fig K. Scatter plot (a) and funnel plot (b) for the MR analysis between WHR and creatine. 13](#_Toc214390093)

[Fig L. Scatter plot (a) and funnel plot (b) for the MR analysis between WHR and tyrosine. 14](#_Toc214390094)

[Fig M. Scatter plot (a) and funnel plot (b) for the MR analysis between WHR and GHCA. 15](#_Toc214390095)

[Fig N. Scatter plot (a) and funnel plot (b) for the MR analysis between WHR and myristoylcarnitine. 16](#_Toc214390096)

[Fig O. Scatter plot (a) and funnel plot (b) for the MR analysis between WHR and arachidonic acid. 17](#_Toc214390097)

[Fig P. Scatter plot (a) and funnel plot (b) for the MR analysis between WHR and 2-hydroxybutyric acid. 18](#_Toc214390098)

[Fig Q Scatter plot (a) and funnel plot (b) for the MR analysis between WHR and pyruvic acid. 19](#_Toc214390099)

[Fig R. Scatter plot (a) and funnel plot (b) for the MR analysis between WHR and hydroxyphenyllactic acid. 20](#_Toc214390100)

[Fig S. Scatter plot (a) and funnel plot (b) for the MR analysis between ABSI_w and DPAn-3. 21](#_Toc214390101)

[Fig T. Scatter plot (a) and funnel plot (b) for the MR analysis between ABSI_w and arachidonic acid. 22](#_Toc214390102)

[Fig U. Scatter plot (a) and funnel plot (b) for the MR analysis between HC and tyrosine. 23](#_Toc214390103)

[Fig V. Scatter plot (a) and funnel plot (b) for the MR analysis between BMI and HCC. 24](#_Toc214390104)

[Fig W. Scatter plot (a) and funnel plot (b) for the MR analysis between WC and HCC. 25](#_Toc214390105)

[Fig X. Scatter plot (a) and funnel plot (b) for the MR analysis between WHR and HCC. 26](#_Toc214390106)

[Fig Y. Scatter plot (a) and funnel plot (b) for the MR analysis between ABSI_w and HCC. 27](#_Toc214390107)


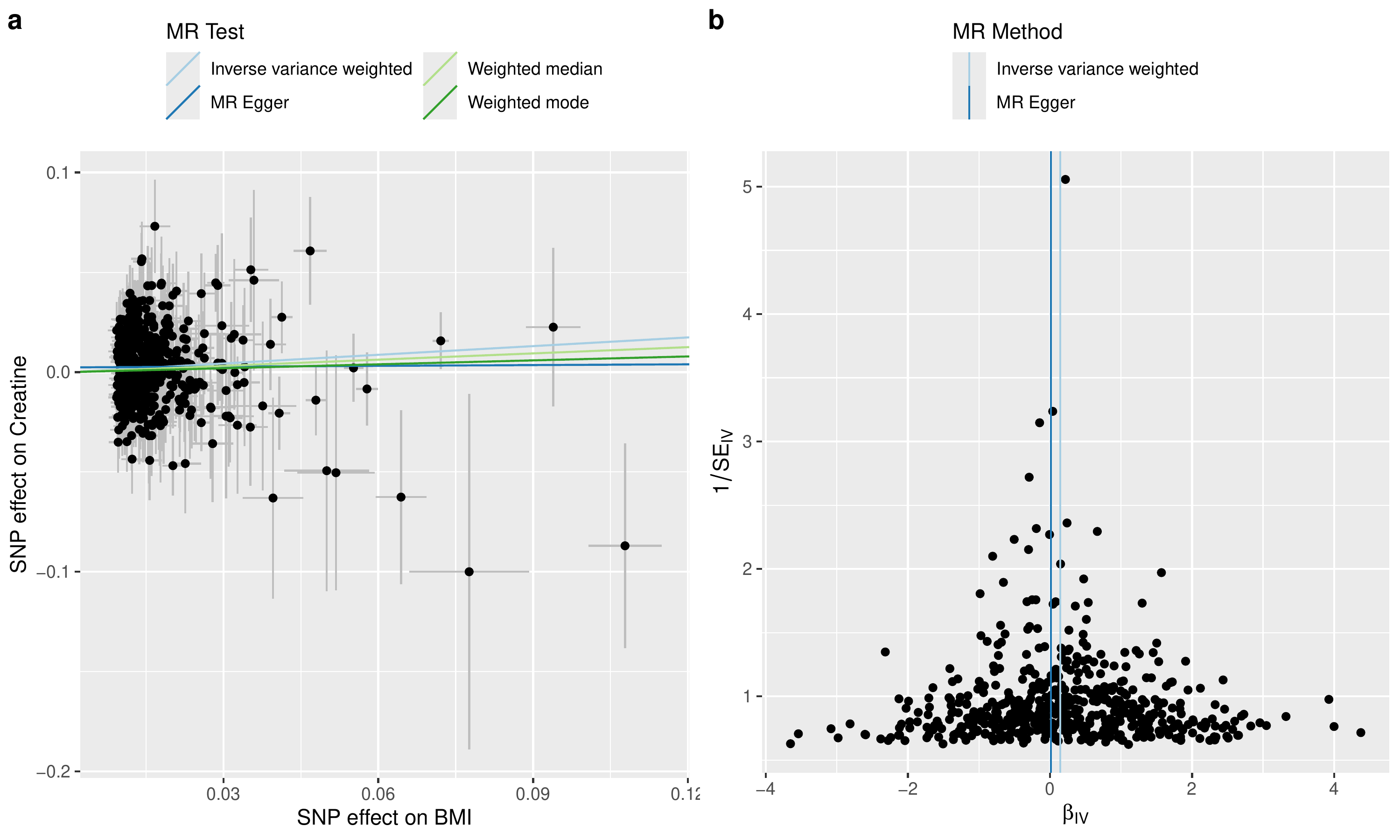


# Fig A. Scatter plot (a) and funnel plot (b) for the MR analysis between BMI and creatine.


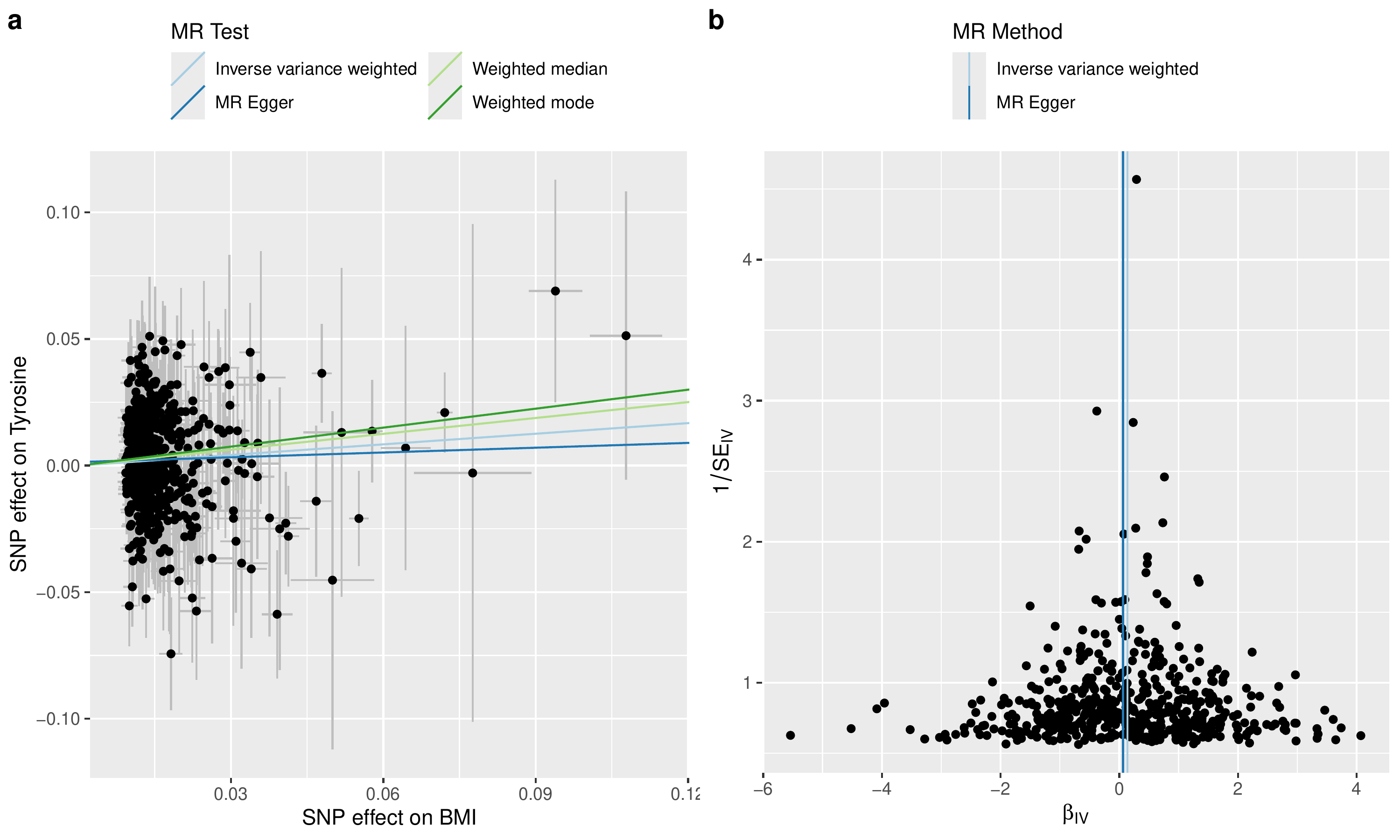


# Fig B. Scatter plot (a) and funnel plot (b) for the MR analysis between BMI and tyrosine.


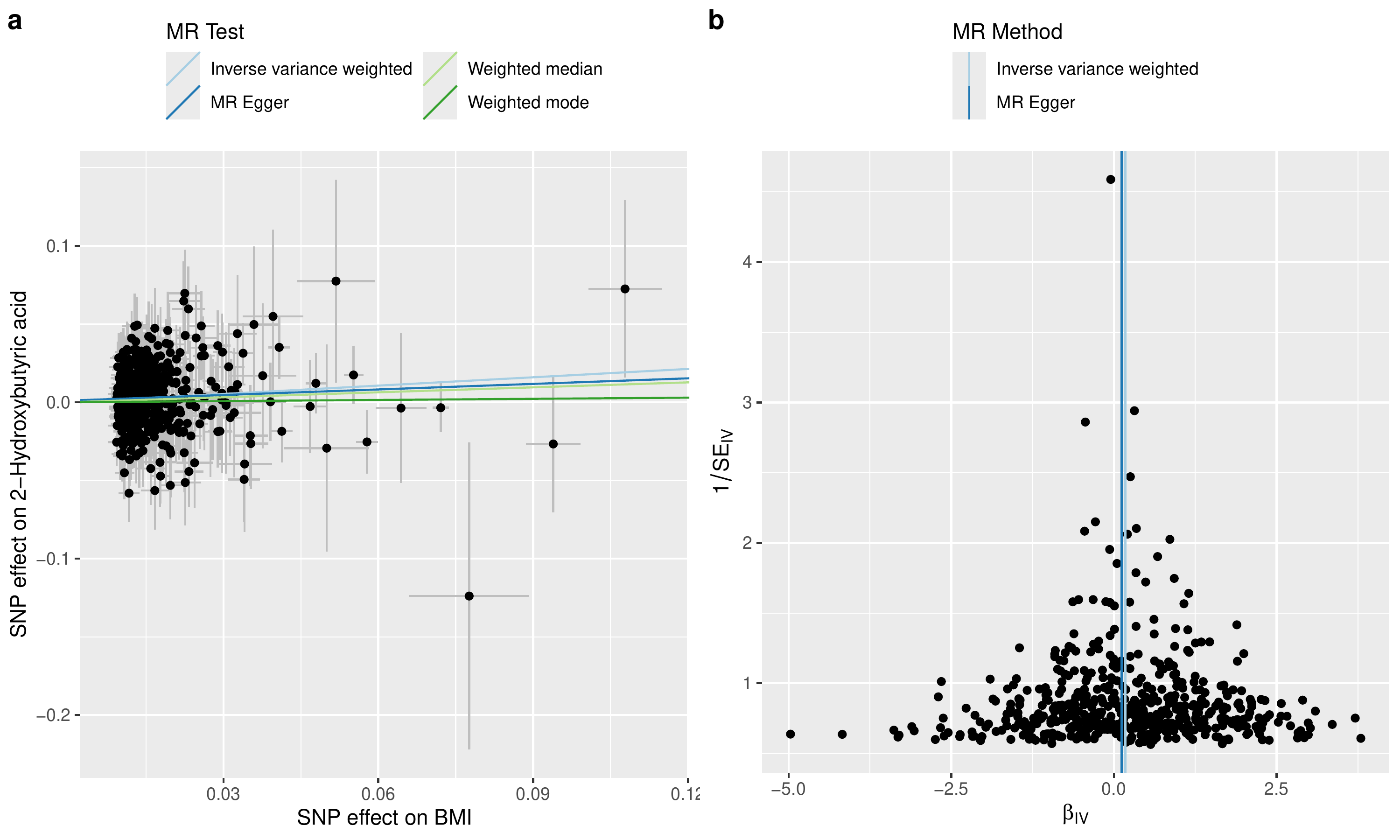


# Fig C. Scatter plot (a) and funnel plot (b) for the MR analysis between BMI and 2-hydroxybutyric acid.


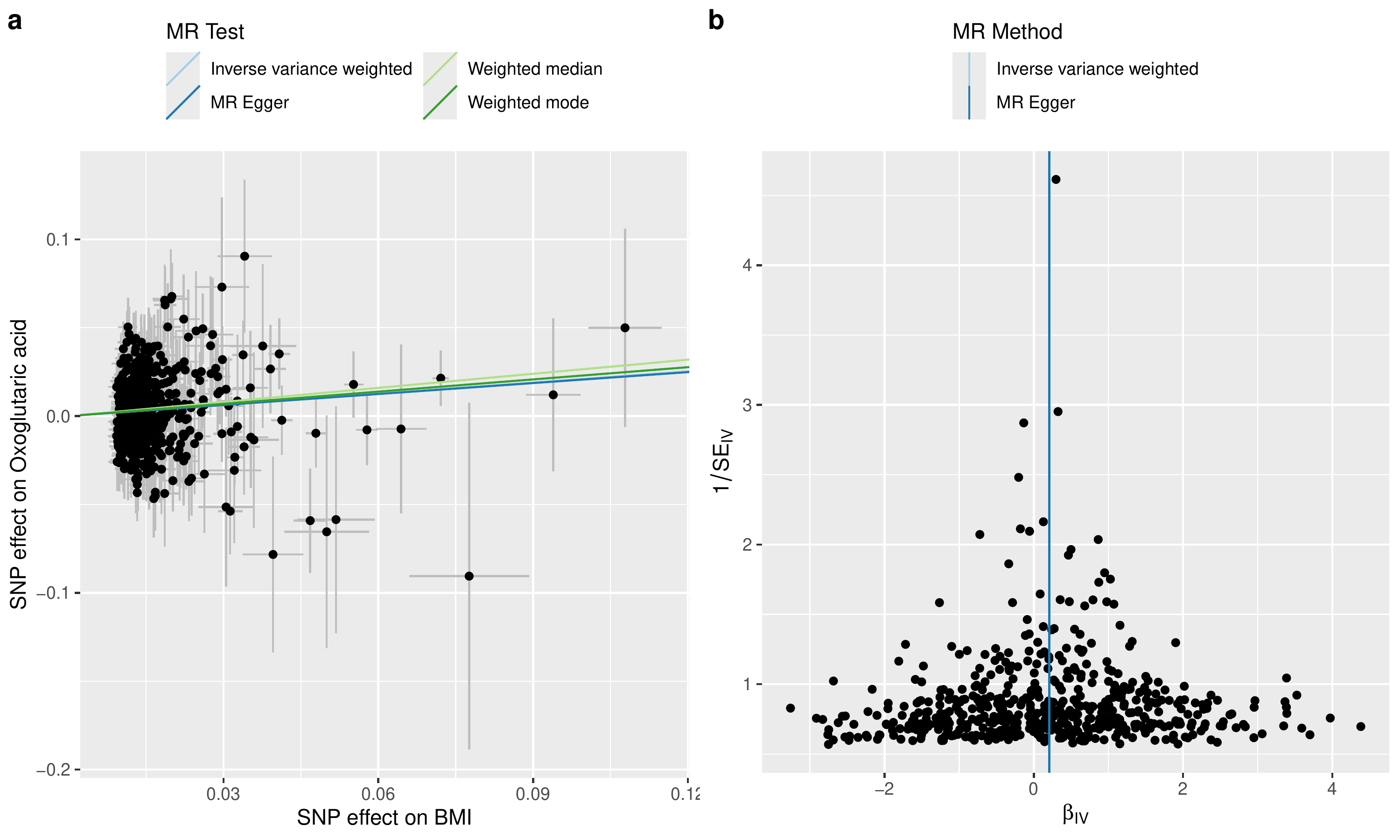


# Fig D. Scatter plot (a) and funnel plot (b) for the MR analysis between BMI and oxoglutaric acid.


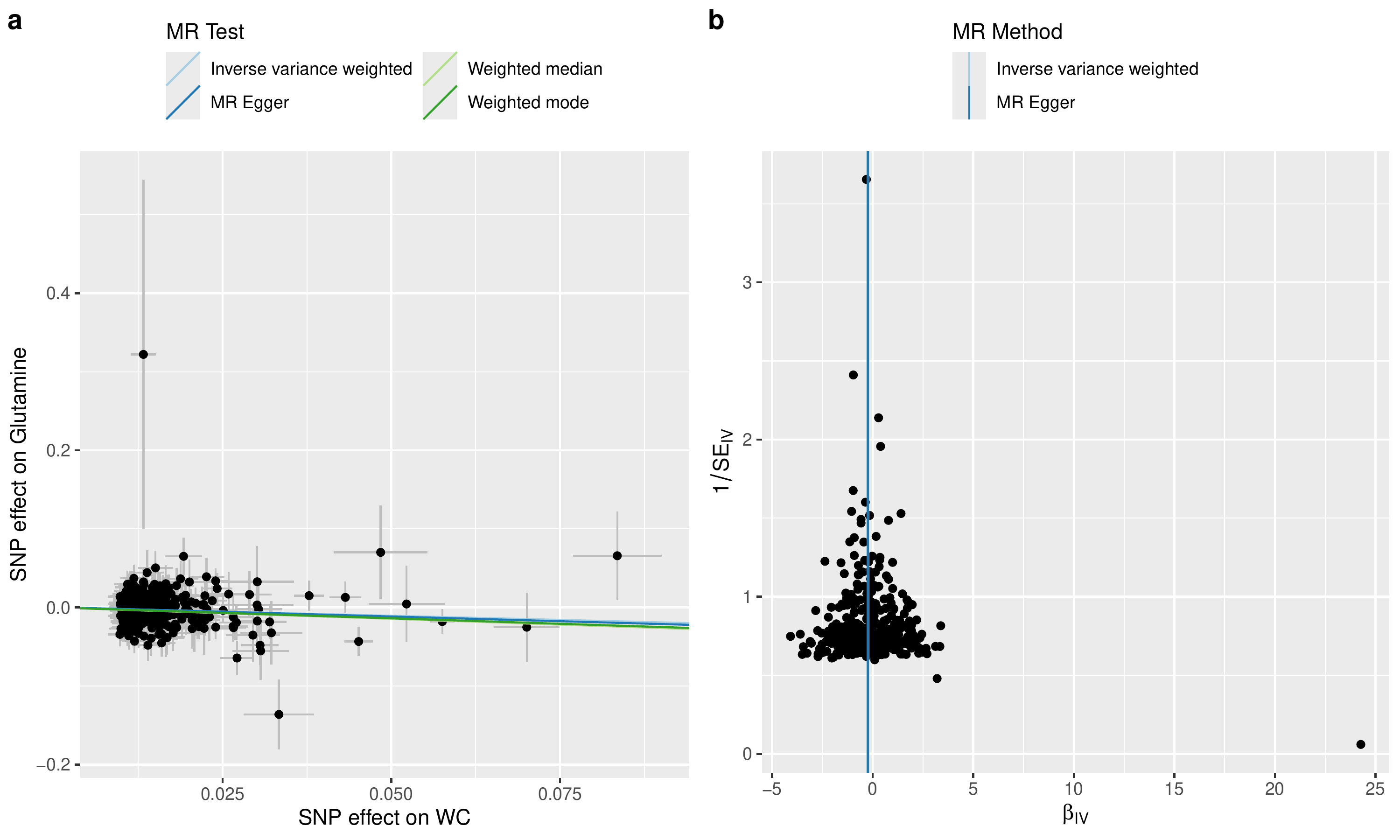


# Fig E. Scatter plot (a) and funnel plot (b) for the MR analysis between WC and glutamine.


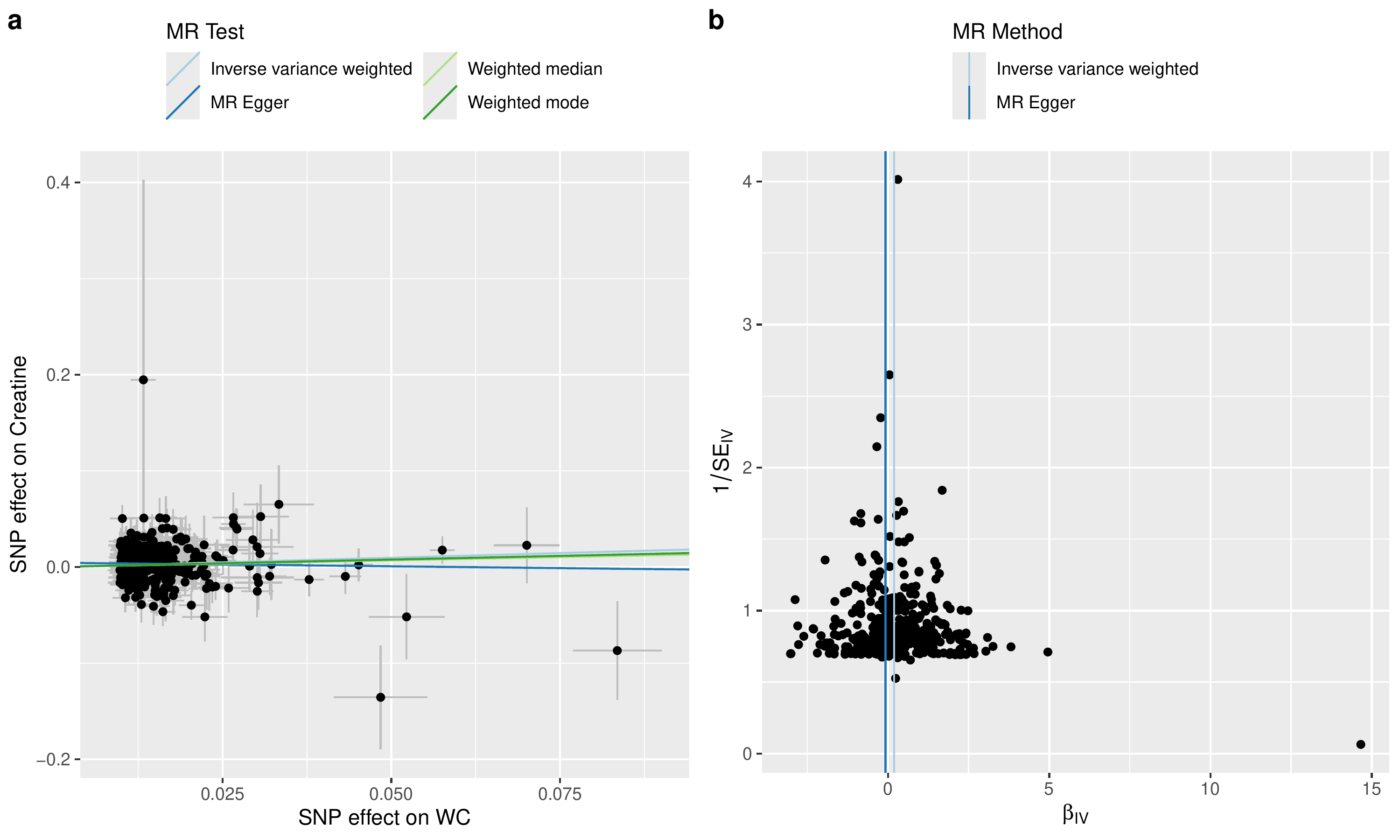


# Fig F. Scatter plot (a) and funnel plot (b) for the MR analysis between WC and creatine.


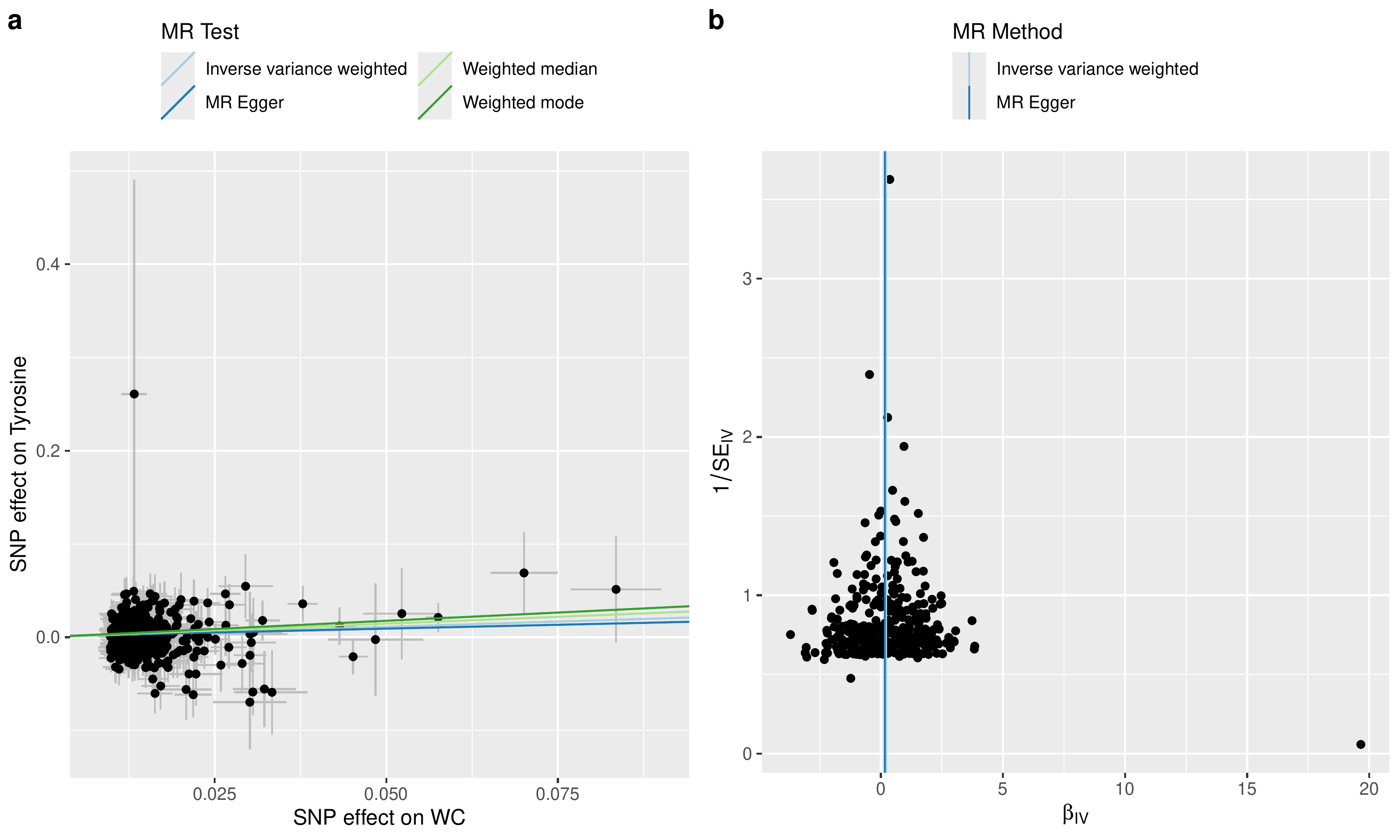


# Fig G. Scatter plot (a) and funnel plot (b) for the MR analysis between WC and tyrosine.


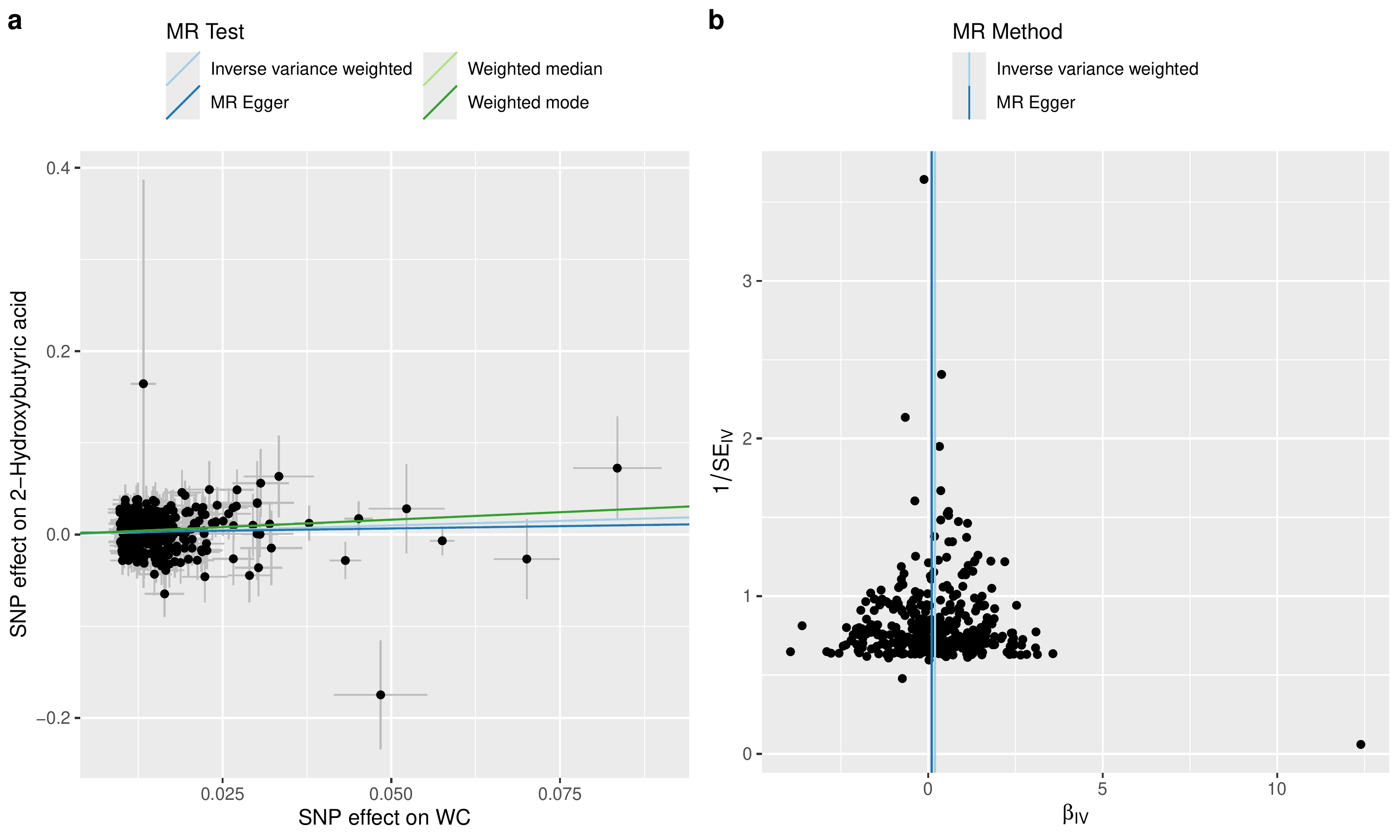


# Fig H. Scatter plot (a) and funnel plot (b) for the MR analysis between WC and 2-hydroxybutyric acid.


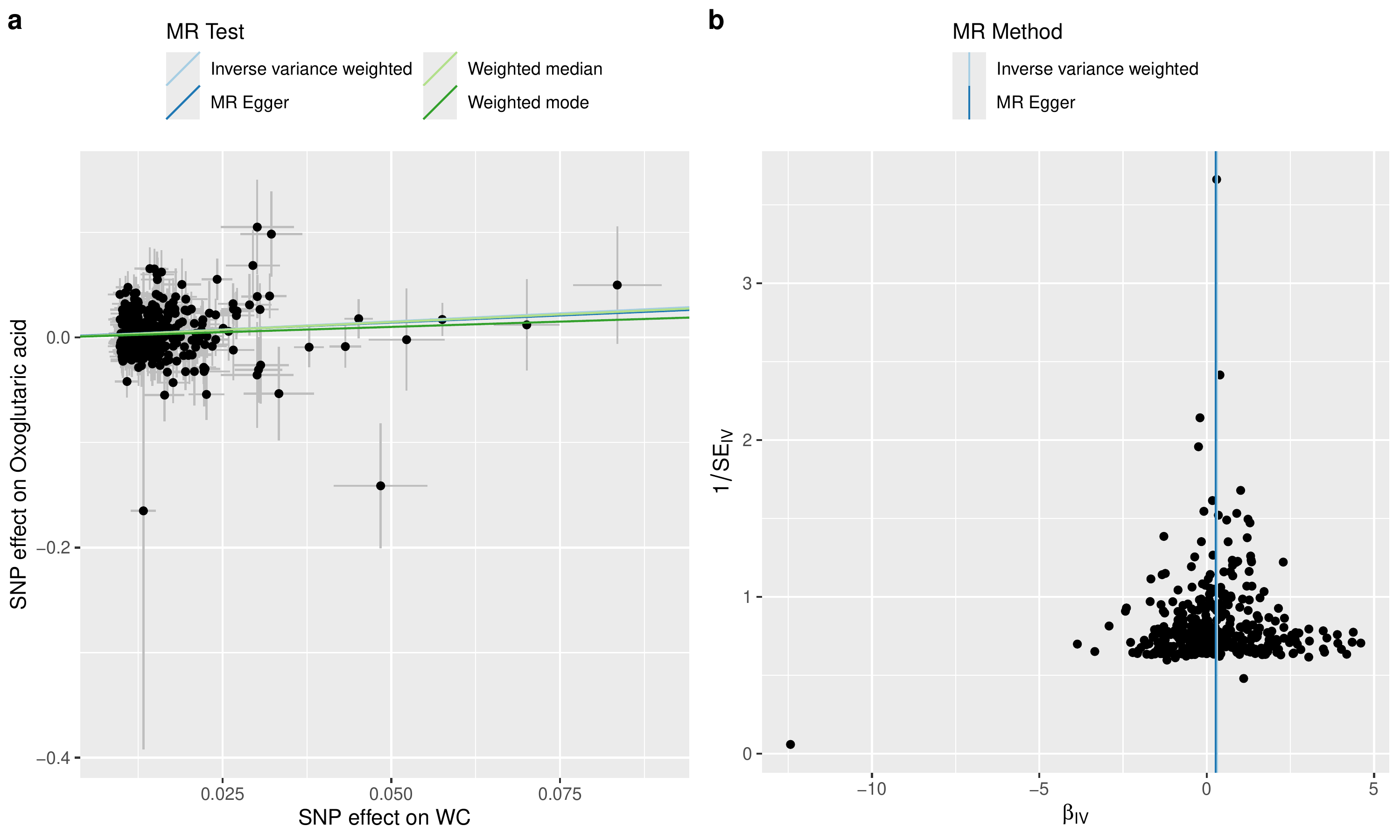


# Fig I. Scatter plot (a) and funnel plot (b) for the MR analysis between WC and oxoglutaric acid.


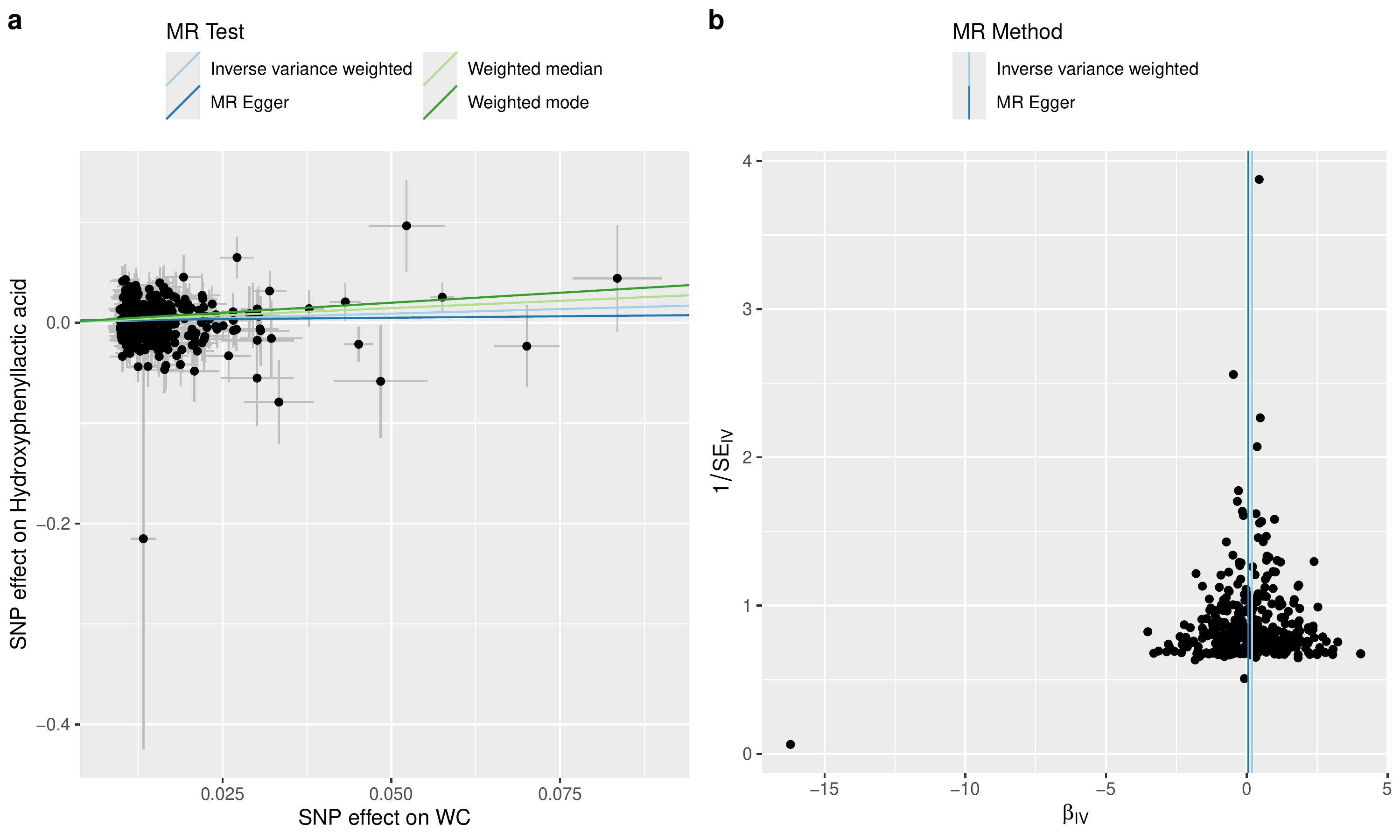


# Fig J. Scatter plot (a) and funnel plot (b) for the MR analysis between WC and hydroxyphenyllactic acid.


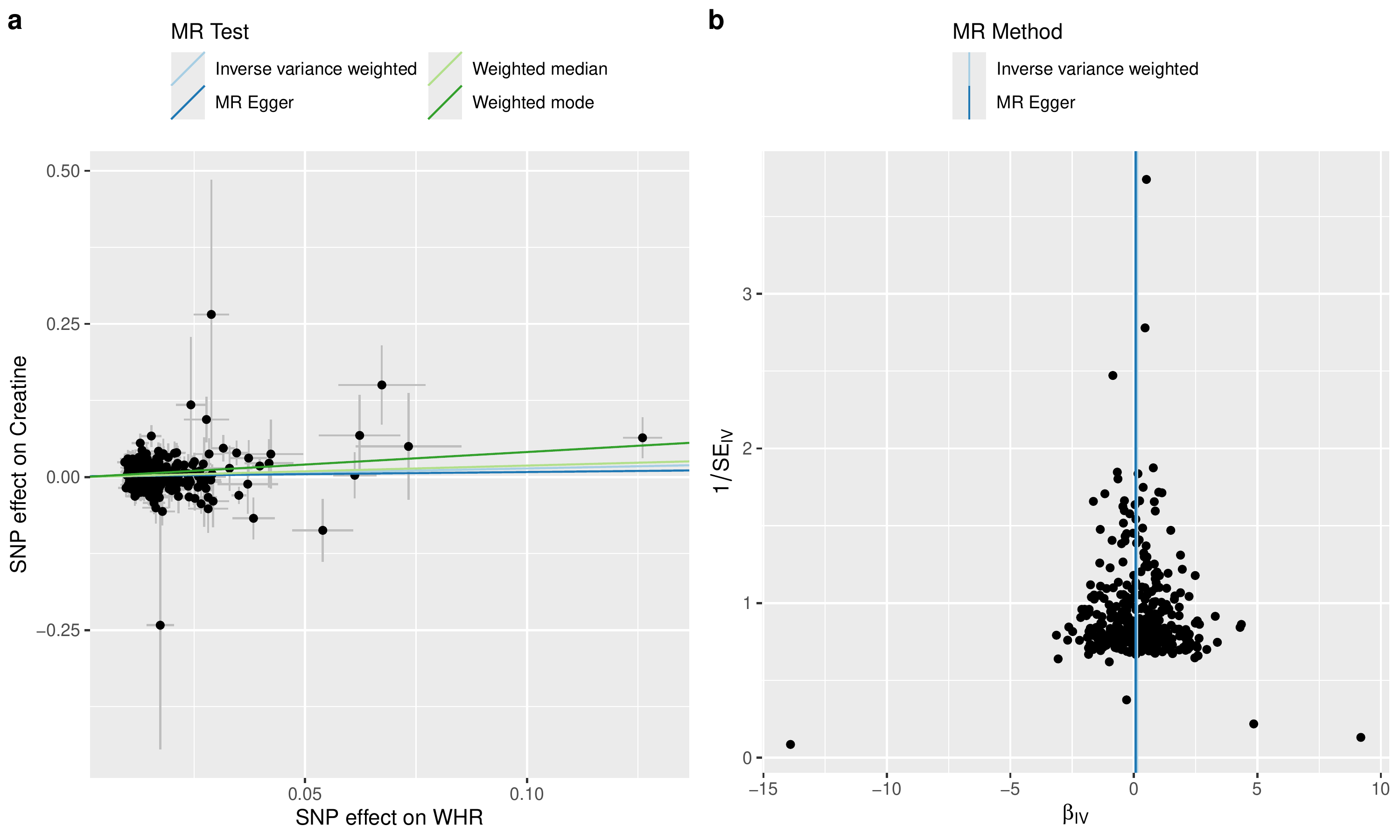


# Fig K. Scatter plot (a) and funnel plot (b) for the MR analysis between WHR and creatine.


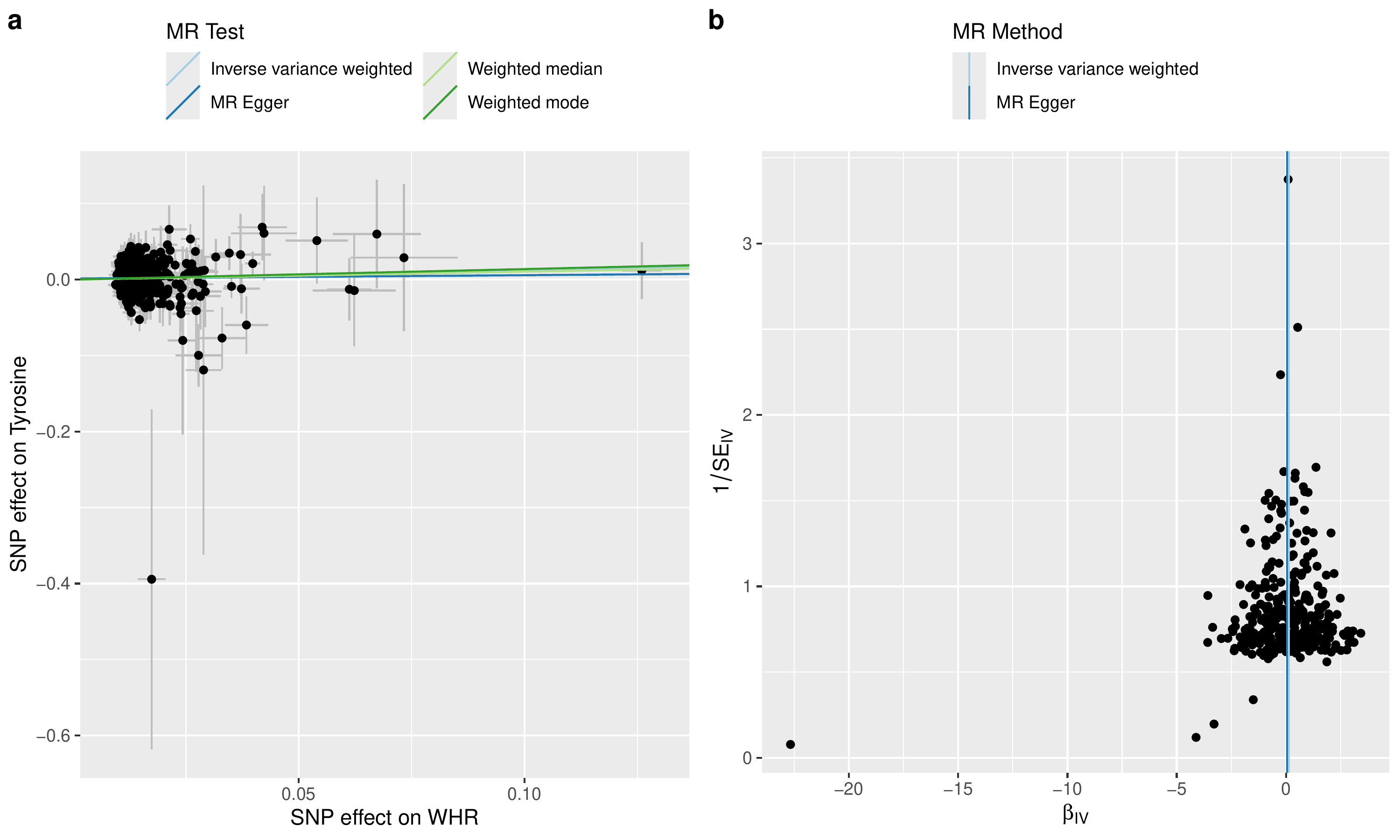


# Fig L. Scatter plot (a) and funnel plot (b) for the MR analysis between WHR and tyrosine.


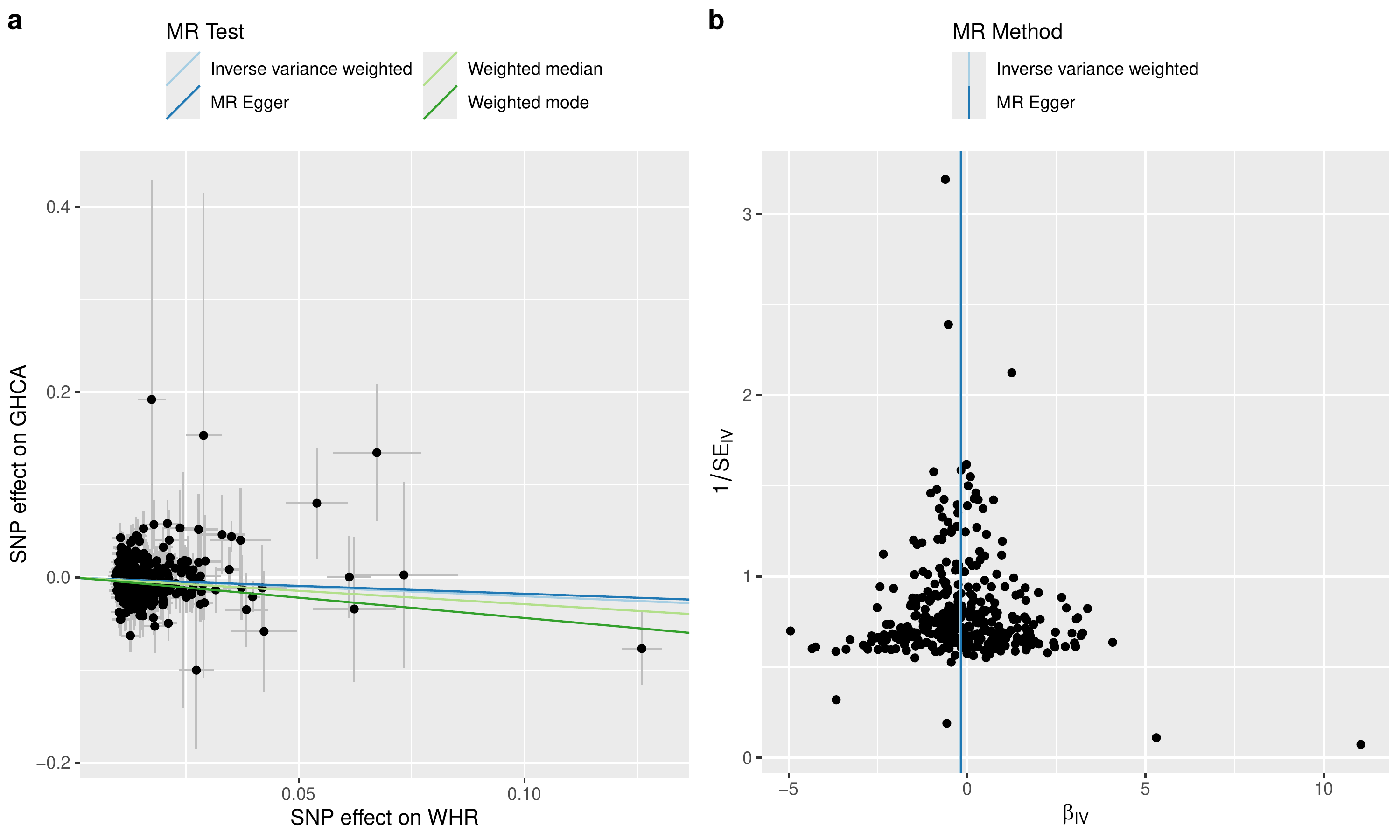


# Fig M. Scatter plot (a) and funnel plot (b) for the MR analysis between WHR and GHCA.


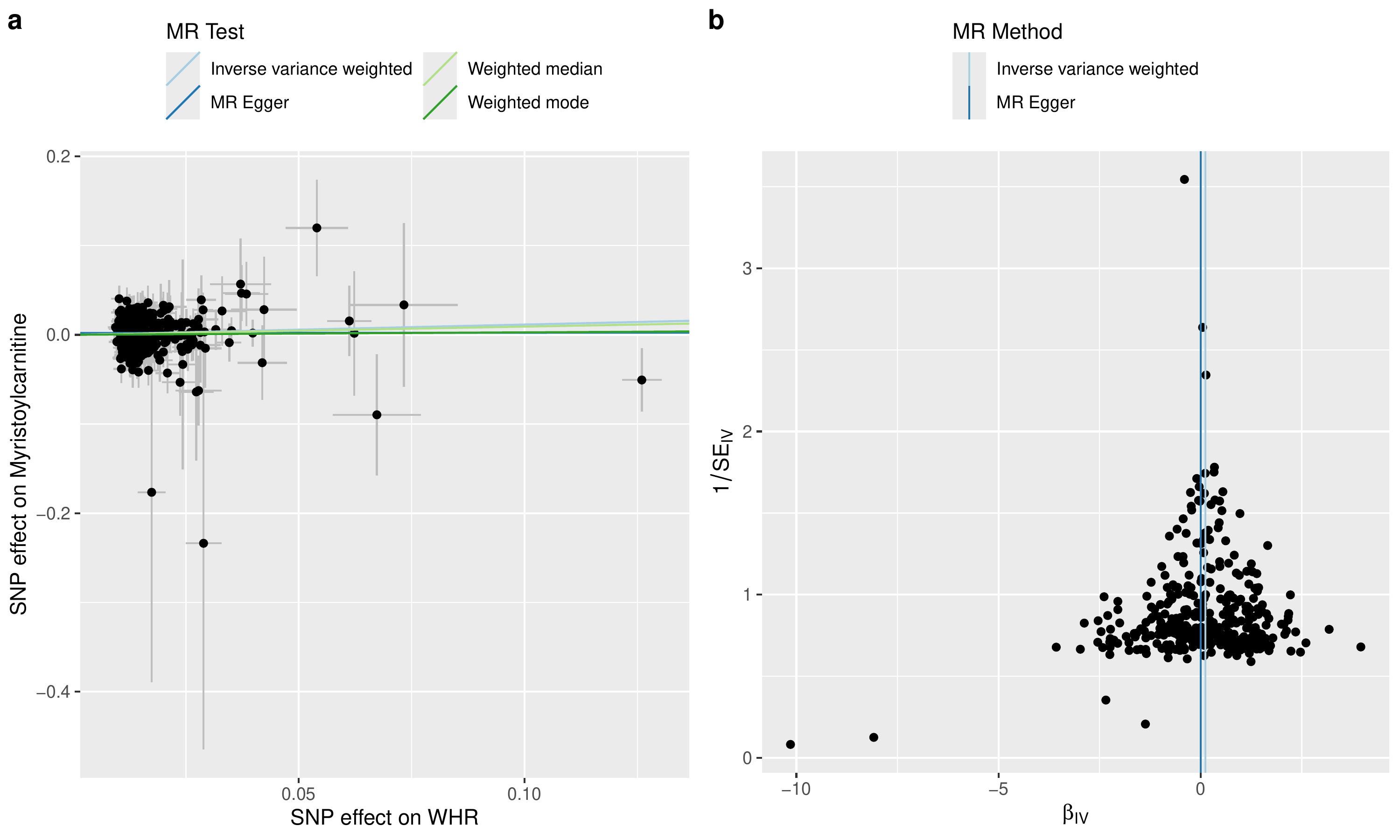


# Fig N. Scatter plot (a) and funnel plot (b) for the MR analysis between WHR and myristoylcarnitine.


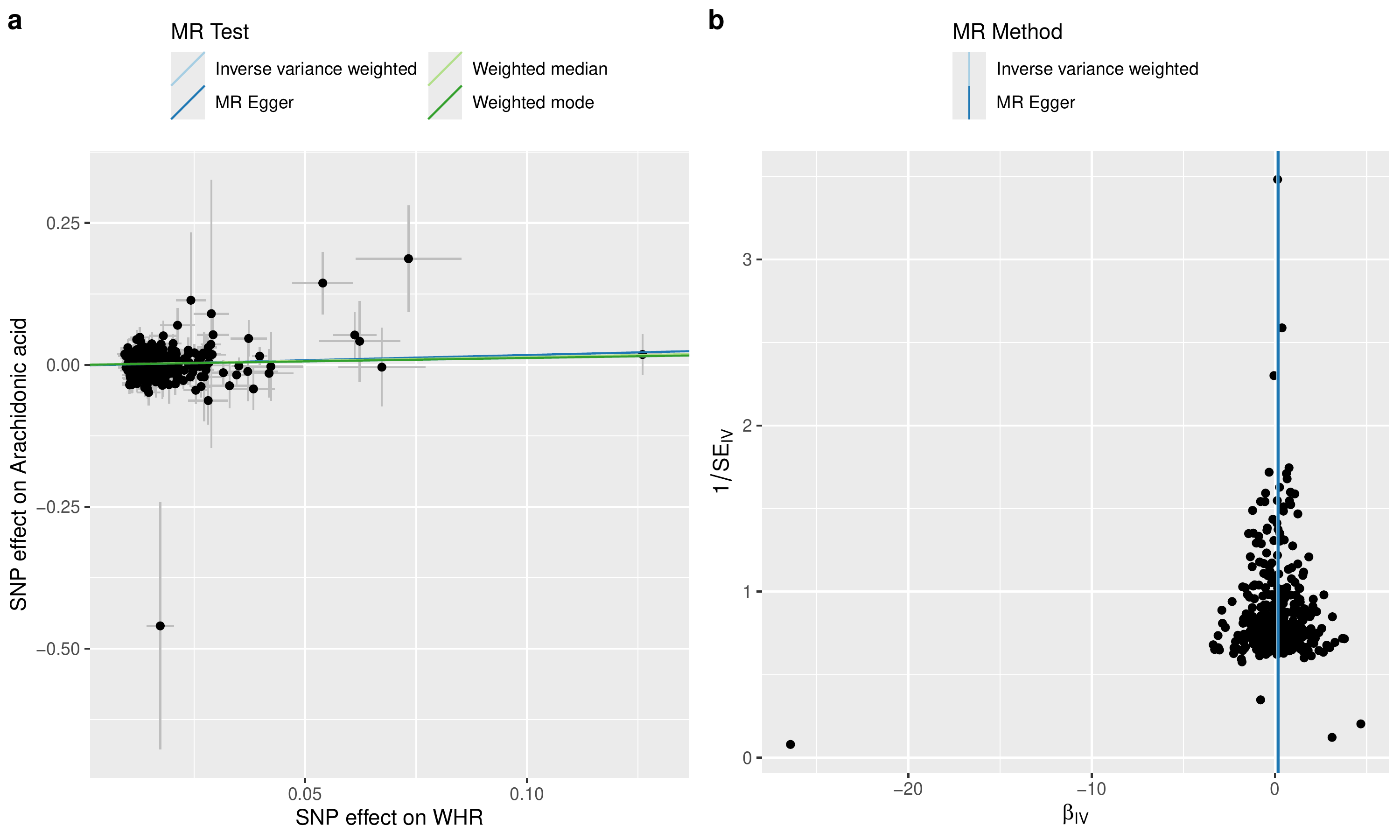


# Fig O. Scatter plot (a) and funnel plot (b) for the MR analysis between WHR and arachidonic acid.


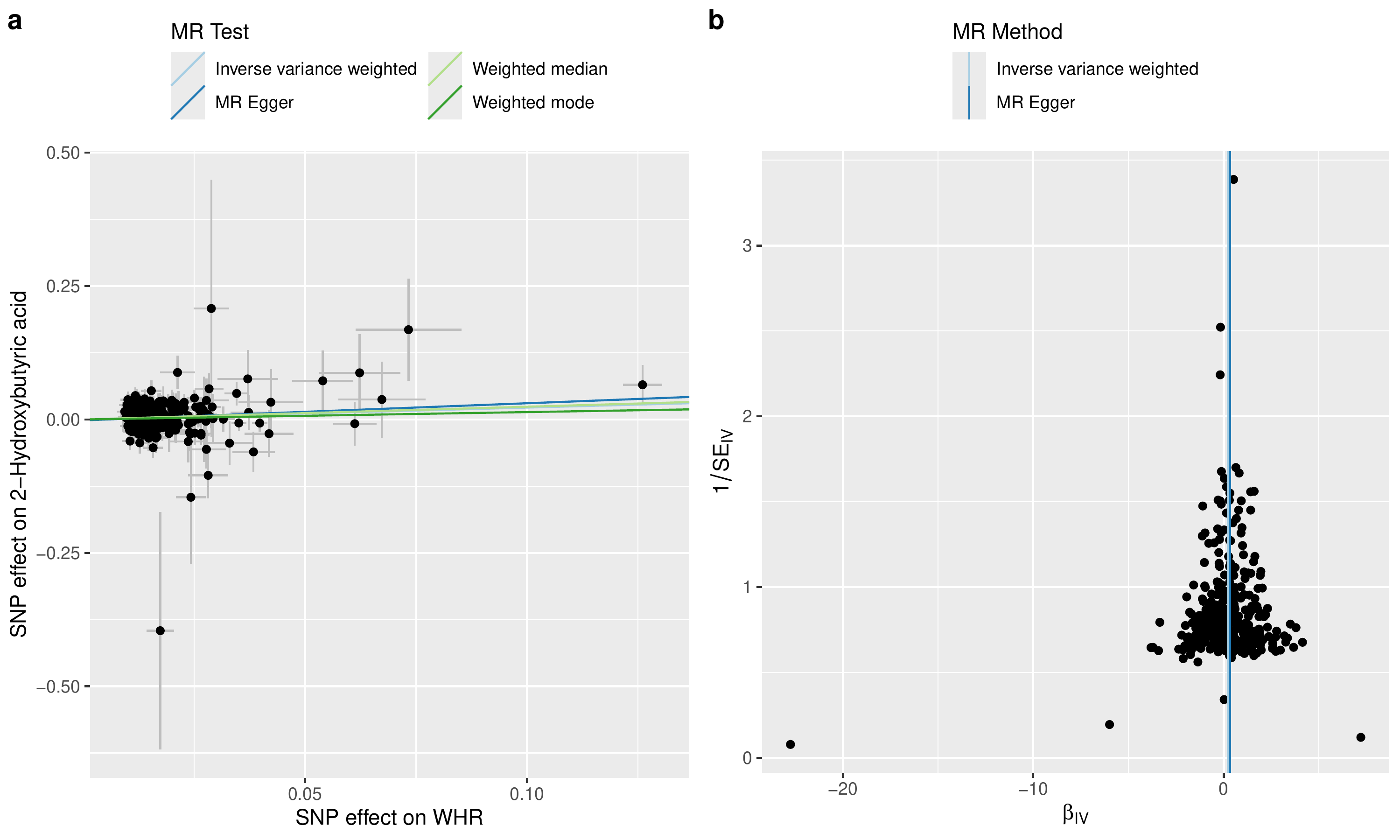


# Fig P. Scatter plot (a) and funnel plot (b) for the MR analysis between WHR and 2-hydroxybutyric acid.


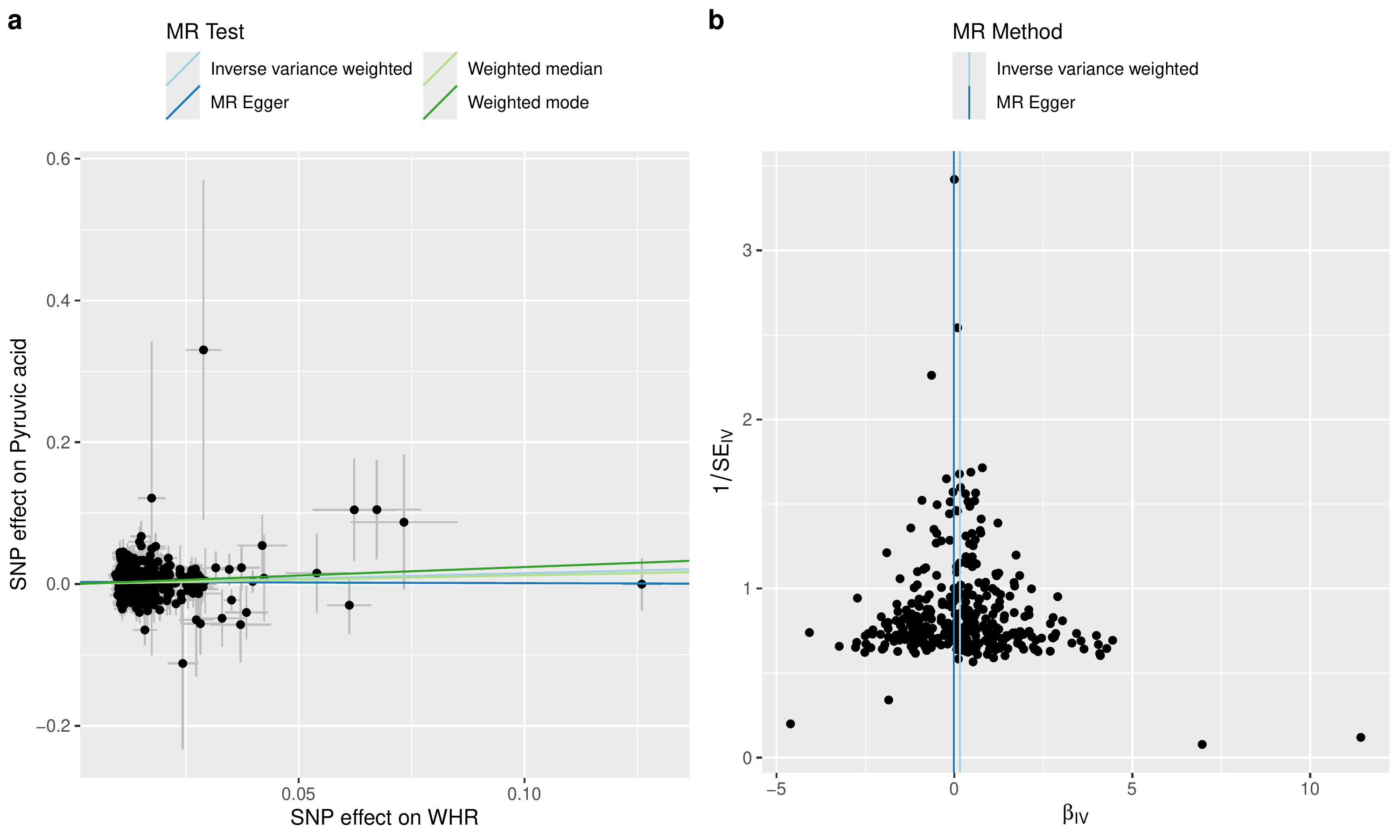


# Fig Q Scatter plot (a) and funnel plot (b) for the MR analysis between WHR and pyruvic acid.


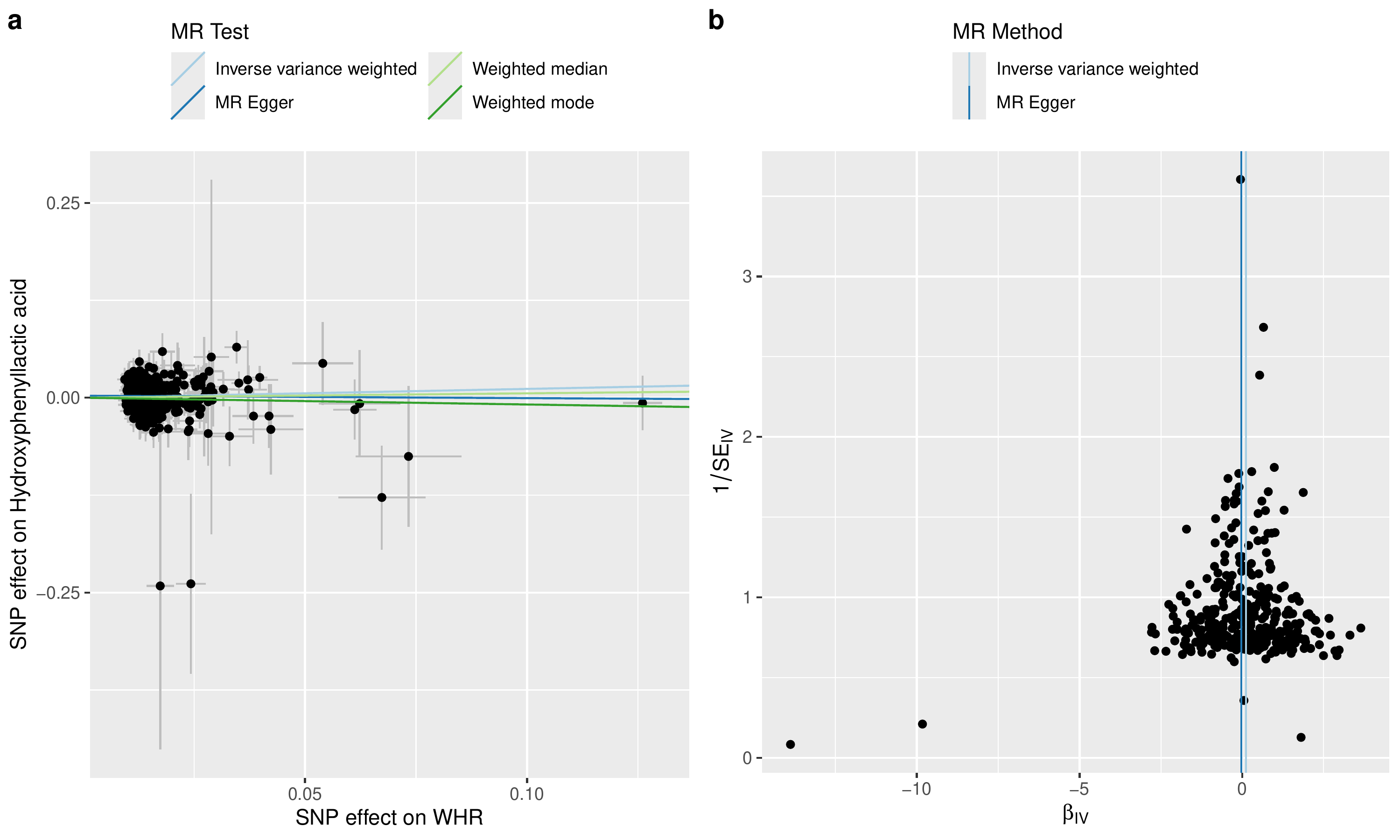


# Fig R. Scatter plot (a) and funnel plot (b) for the MR analysis between WHR and hydroxyphenyllactic acid.


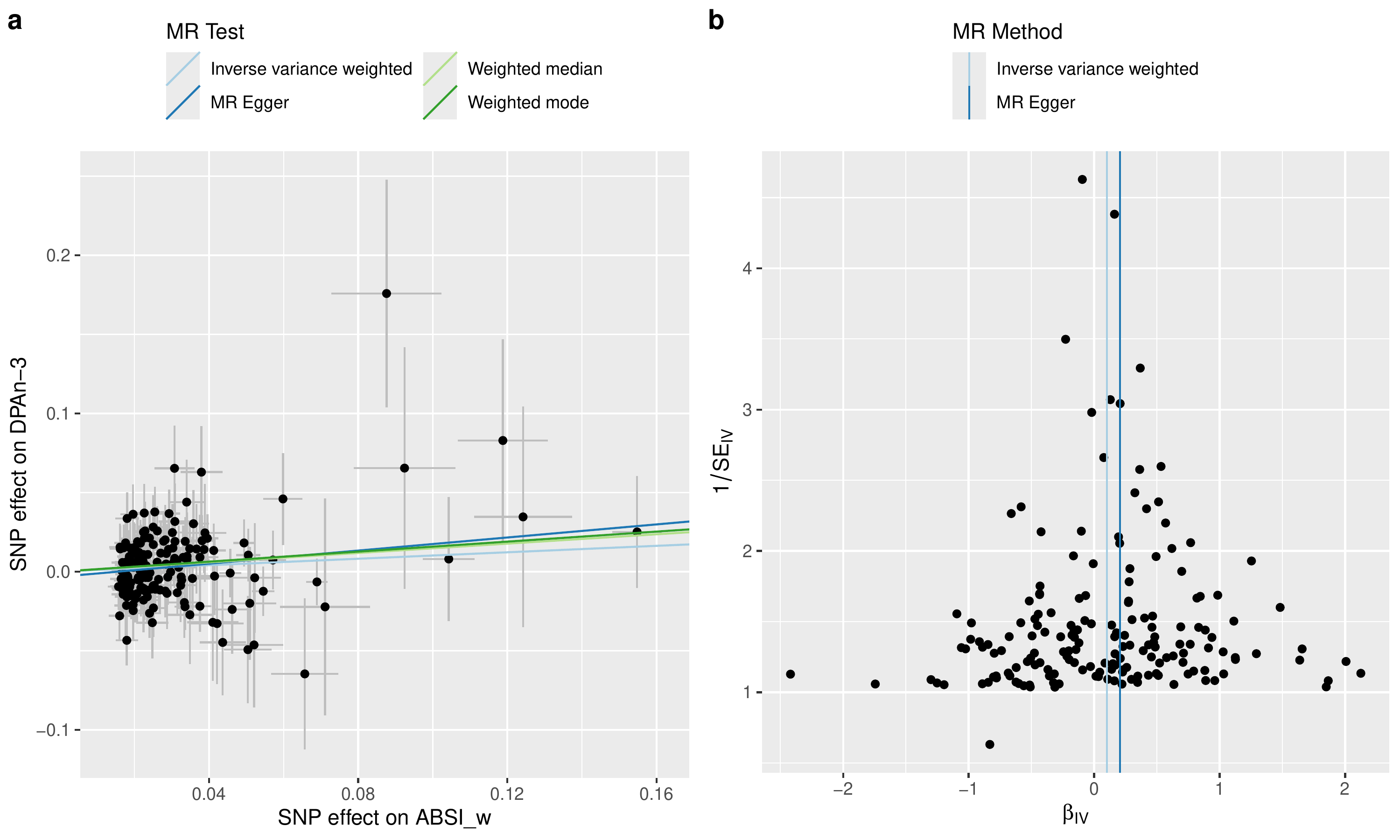


# Fig S. Scatter plot (a) and funnel plot (b) for the MR analysis between ABSI_w and DPAn-3.


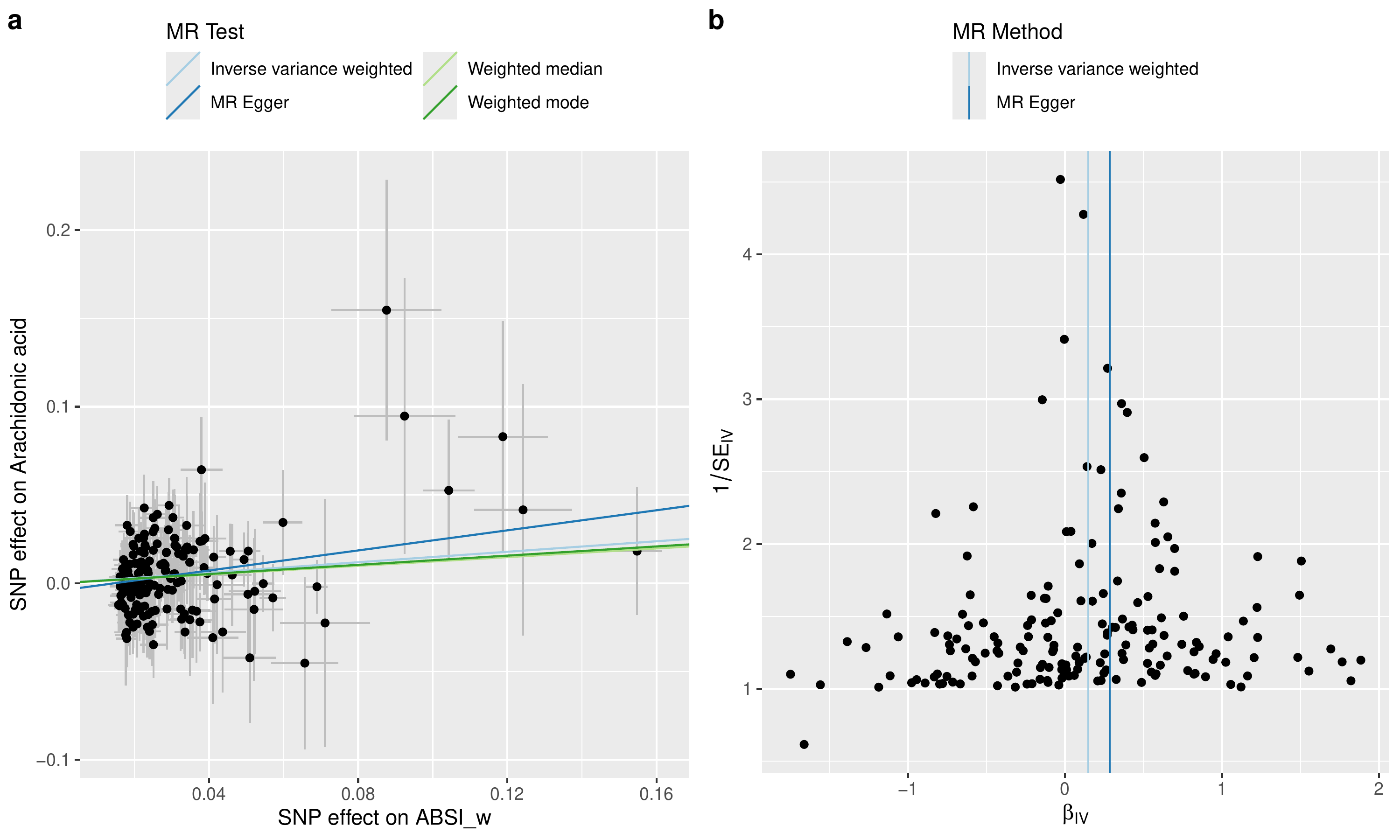


# Fig T. Scatter plot (a) and funnel plot (b) for the MR analysis between ABSI_w and arachidonic acid.


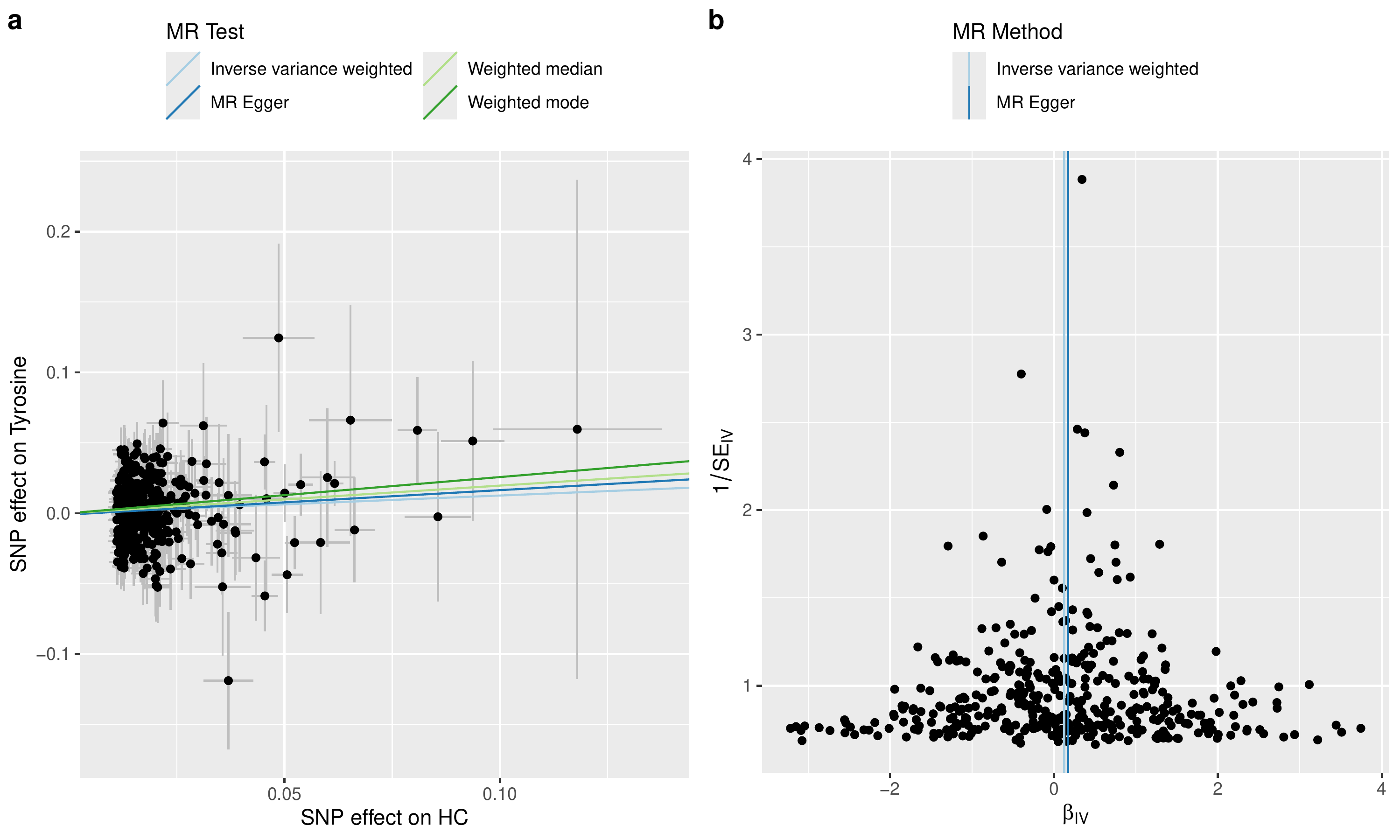


# Fig U. Scatter plot (a) and funnel plot (b) for the MR analysis between HC and tyrosine.


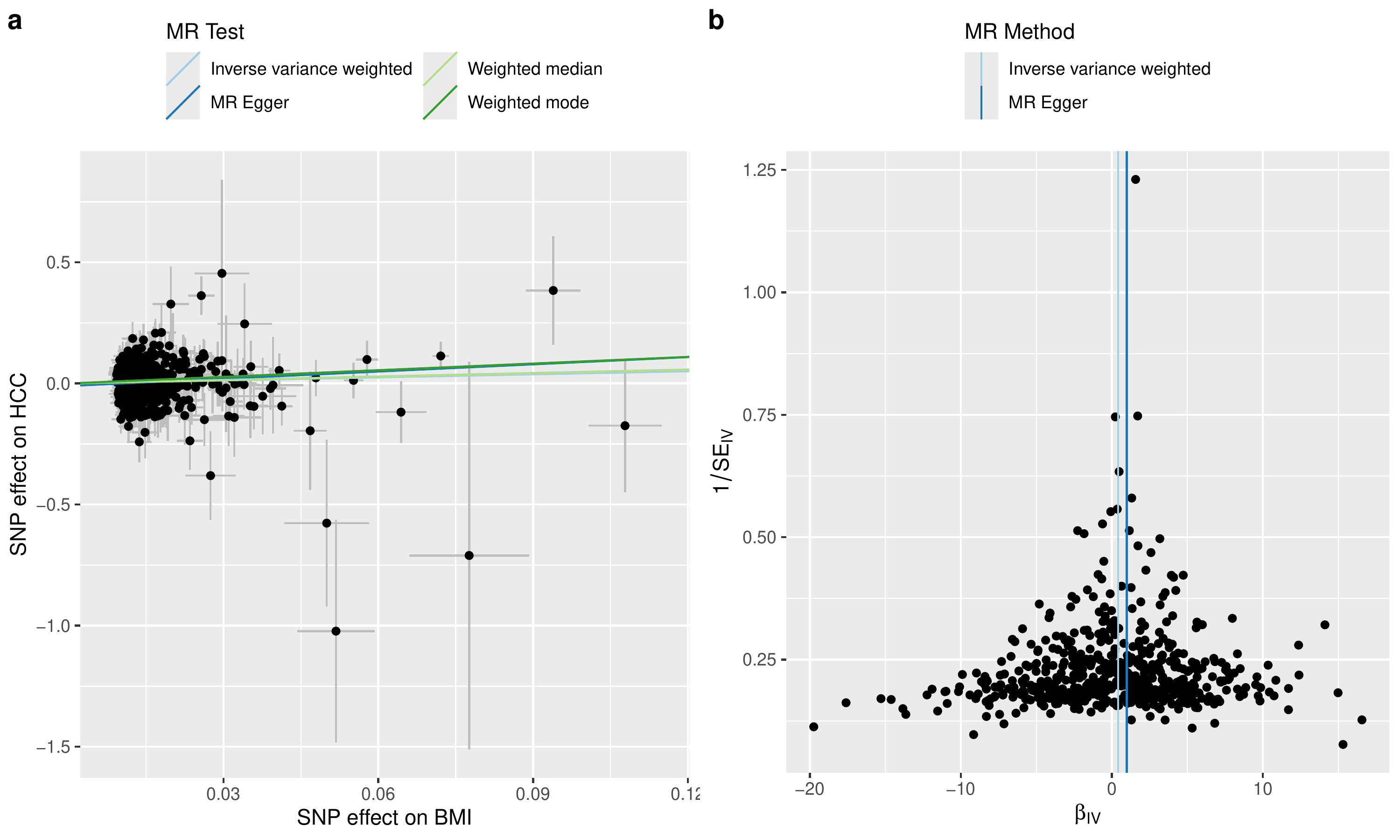


# Fig V. Scatter plot (a) and funnel plot (b) for the MR analysis between BMI and HCC.


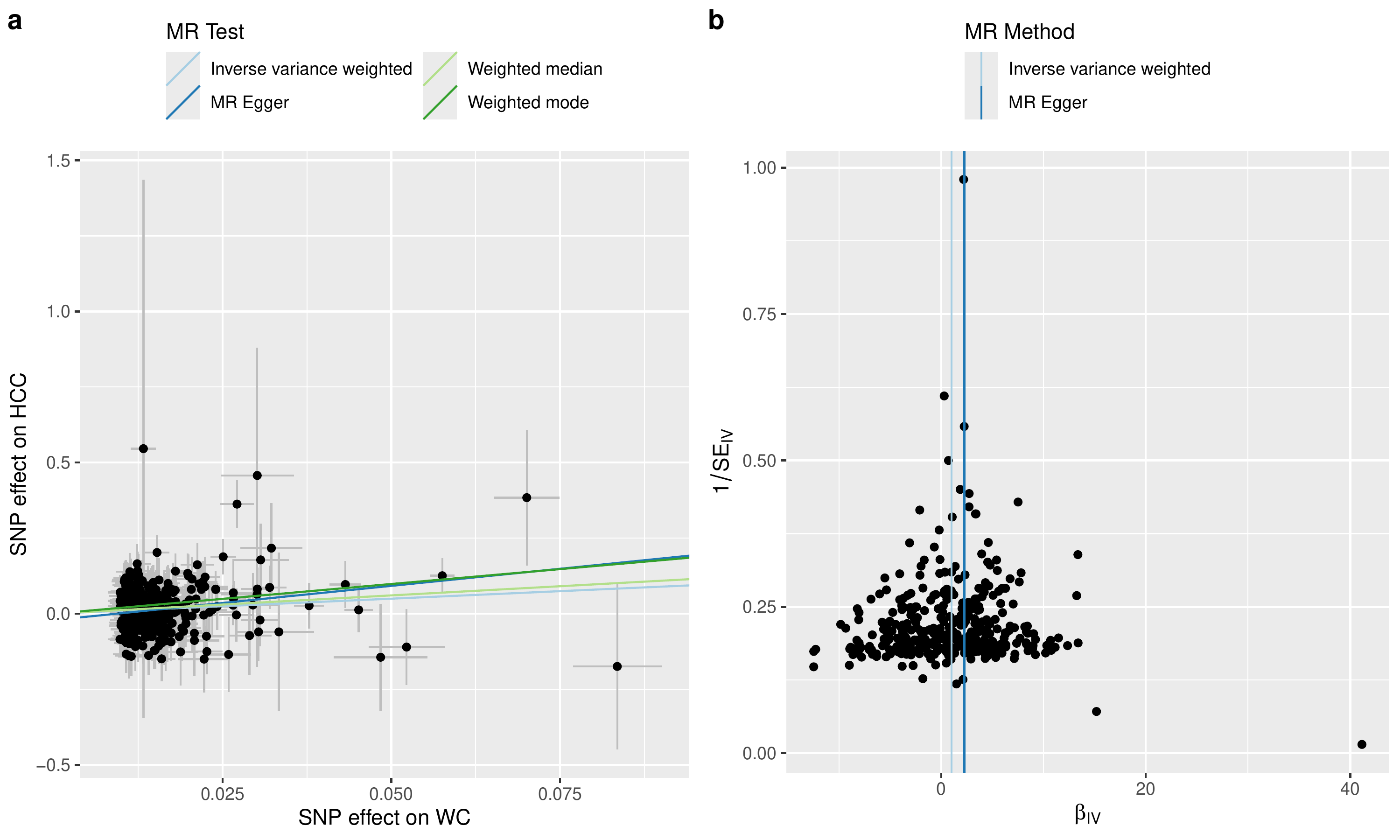


# Fig W. Scatter plot (a) and funnel plot (b) for the MR analysis between WC and HCC.


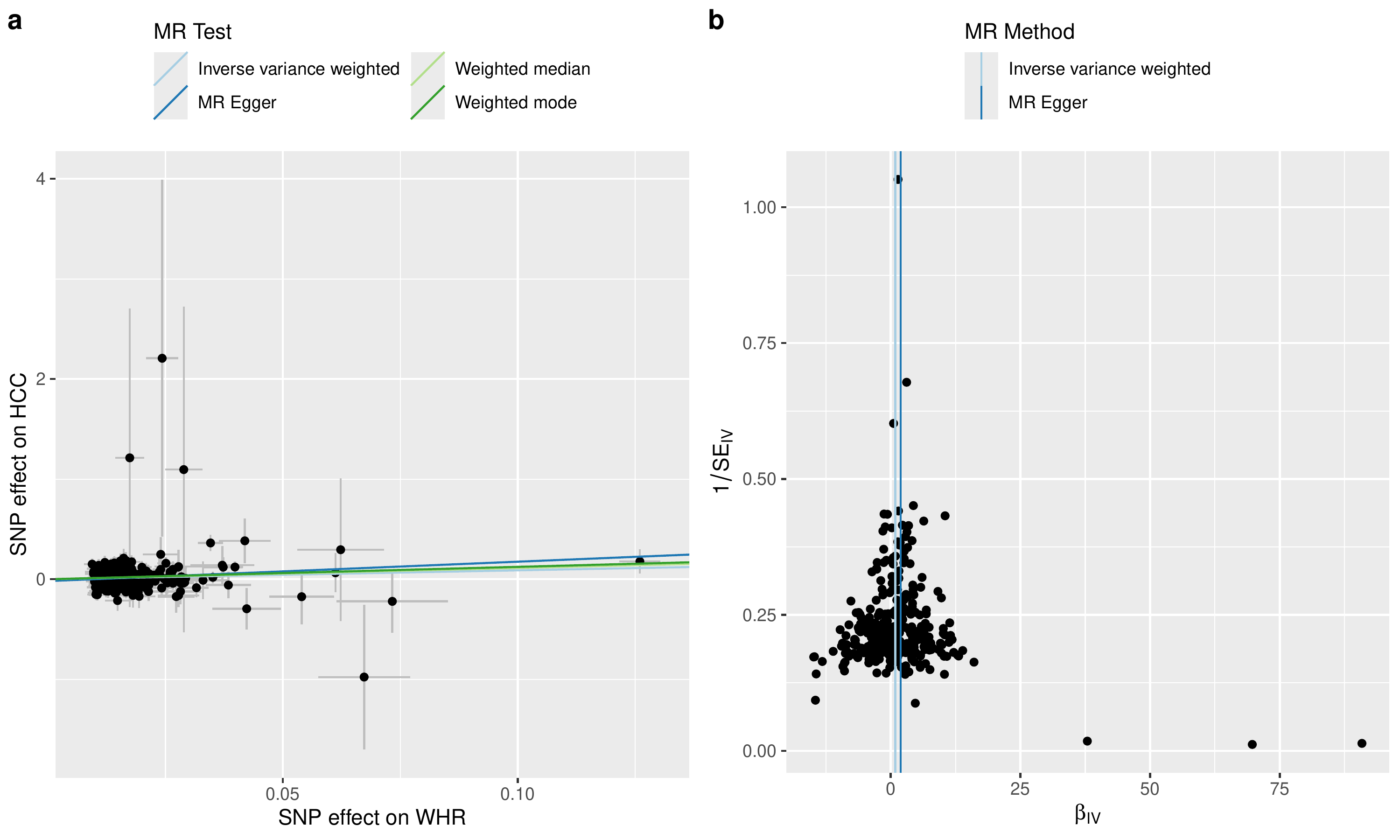


# Fig X. Scatter plot (a) and funnel plot (b) for the MR analysis between WHR and HCC.


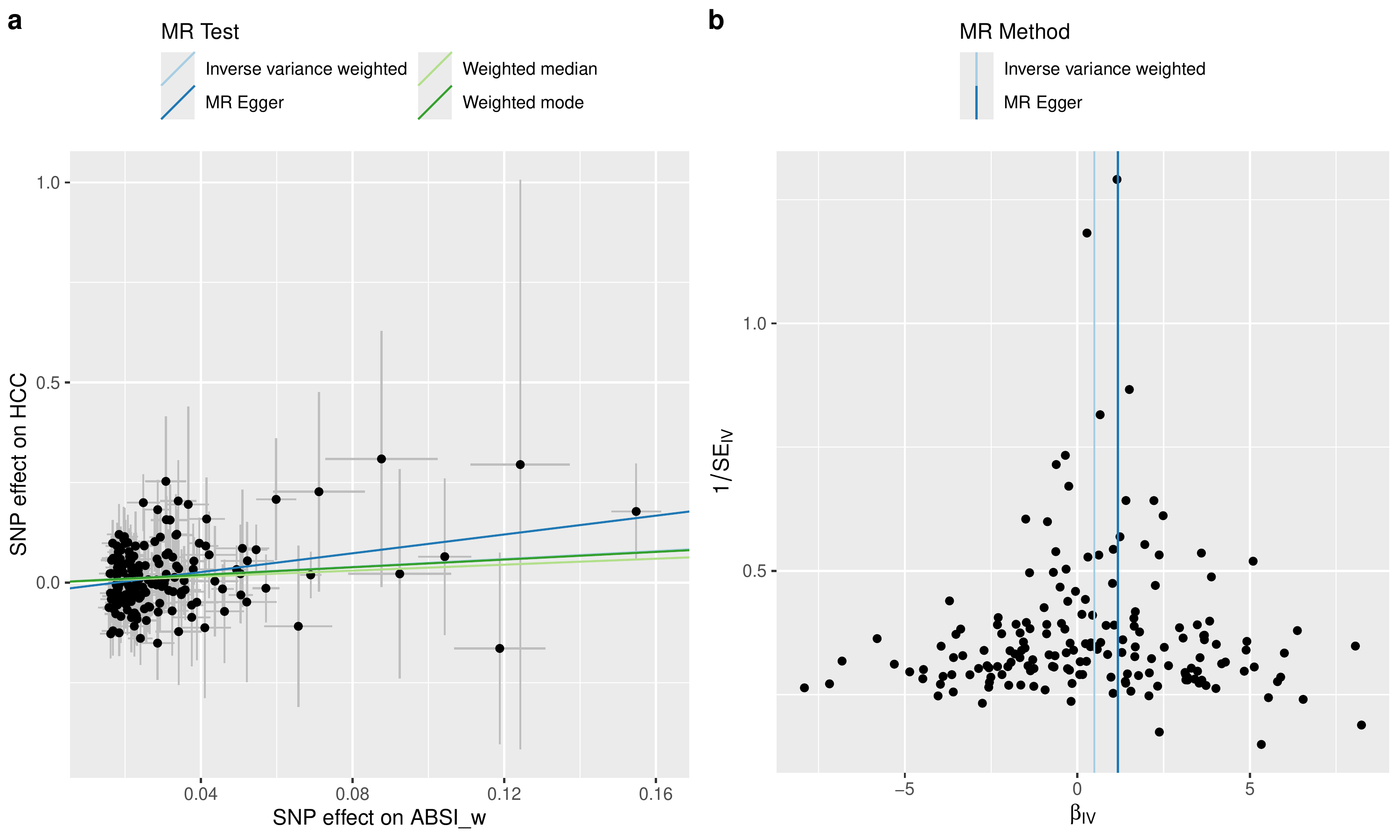


# Fig Y. Scatter plot (a) and funnel plot (b) for the MR analysis between ABSI_w and HCC.
